# Anthocyanins and Their Metabolites as Therapeutic Agents for Neurodegenerative Disease

**DOI:** 10.3390/antiox8090333

**Published:** 2019-08-22

**Authors:** Aimee N. Winter, Paula C. Bickford

**Affiliations:** 1 Department of Neurosurgery and Brain Repair, Morsani College of Medicine, University of South Florida Health, 12901 Bruce B Downs Blvd, Tampa, FL 33612, USA; 2 Department of Molecular Pharmacology and Physiology, Morsani College of Medicine, University of South Florida Health, 12901 Bruce B Downs Blvd, Tampa, FL 33612, USA; 3 Research Service, James A Haley Veterans Hospital, 13000 Bruce B Downs Blvd, Tampa, FL 33612, USA

**Keywords:** anthocyanins, phenolic acids, flavonoids, neurodegeneration, Alzheimer’s disease, Parkinson’s disease, amyotrophic lateral sclerosis, oxidative stress, inflammation, neuroprotection

## Abstract

Neurodegenerative diseases, including Alzheimer’s disease, Parkinson’s disease, and amyotrophic lateral sclerosis (ALS), are characterized by the death of neurons within specific regions of the brain or spinal cord. While the etiology of many neurodegenerative diseases remains elusive, several factors are thought to contribute to the neurodegenerative process, such as oxidative and nitrosative stress, excitotoxicity, endoplasmic reticulum stress, protein aggregation, and neuroinflammation. These processes culminate in the death of vulnerable neuronal populations, which manifests symptomatically as cognitive and/or motor impairments. Until recently, most treatments for these disorders have targeted single aspects of disease pathology; however, this strategy has proved largely ineffective, and focus has now turned towards therapeutics which target multiple aspects underlying neurodegeneration. Anthocyanins are unique flavonoid compounds that have been shown to modulate several of the factors contributing to neuronal death, and interest in their use as therapeutics for neurodegeneration has grown in recent years. Additionally, due to observations that the bioavailability of anthocyanins is low relative to that of their metabolites, it has been proposed that anthocyanin metabolites may play a significant part in mediating the beneficial effects of an anthocyanin-rich diet. Thus, in this review, we will explore the evidence evaluating the neuroprotective and therapeutic potential of anthocyanins and their common metabolites for treating neurodegenerative diseases.

## 1. Introduction

With recent advances in science and medicine, the world has experienced a steady rise in average life expectancy and a concomitant rise in the incidence of neurodegenerative diseases. Moreover, since neurodegenerative diseases strike predominately in mid- to late-life, the number of individuals with disorders like Alzheimer’s disease, Parkinson’s disease, and amyotrophic lateral sclerosis (ALS) is expected to keep climbing in years to come. Unfortunately, despite the rising incidence of these diseases, developing therapeutic strategies for treating neurodegeneration has proved challenging, and current treatments remain largely palliative.

**Figure 1:**
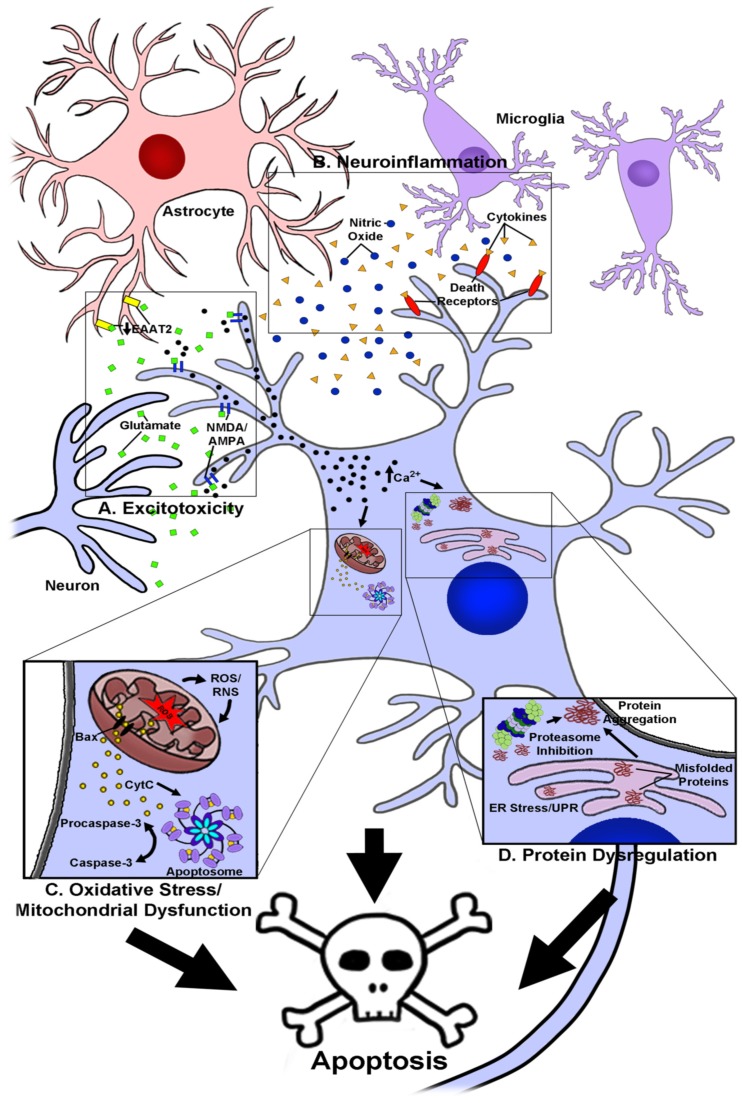
Molecular mechanisms contributing to the pathogenesis of neurodegenerative diseases. **A**, The role of excitotoxicity in neurodegeneration. Glutamate is released from pre-synaptic neuron terminals in elevated quantities and binds to glutamate receptors such as *N*-methyl-d-aspartate (NMDA) and α-amino-3-hydroxy-5-methyl-4-isoxazolepropionic acid (AMPA) receptors. Receptor binding causes massive calcium influx in post-synaptic neurons and activates pro-apoptotic signaling cascades, in addition to inducing mitochondrial dysfunction and endoplasmic reticulum (ER) stress responses. In some diseases, such as ALS, glutamate uptake from the synapse by the excitatory amino acid transporter 2 (EAAT2) in astrocytes is impaired, exacerbating glutamate excitotoxicity. **B**, The role of neuroinflammation in neurodegeneration. Glial cells such as astrocytes and microglia become chronically enflamed in disease states and secrete oxidative species, such as nitric oxide, and pro-inflammatory cytokines. Cytokines bind to death receptors on the cell surface and activate pro-apoptotic signaling cascades. **C**, The role of oxidative stress and mitochondrial dysfunction in neurodegeneration. Mitochondrial dysfunction occurs as a result of several factors, causing mitochondria to produce elevated levels of reactive oxygen and nitrogen species (ROS and RNS). Enhanced production of ROS and RNS exacerbates mitochondrial dysfunction, eventually causing release of the pro-apoptotic signaling protein, cytochrome c (CytC). Cytochrome C contributes to formation of the apoptosome, which in turn cleaves procaspase-3 to form active caspase-3, stimulating apoptosis. **D**, The role of protein dysregulation in neurodegeneration. ER stress occurs as a result of multiple factors, such as the presence of mutated or damaged proteins, causing accumulation of misfolded proteins and activation of the unfolded protein response (UPR). Misfolded or mutated proteins are trafficked to the proteasome for degradation. As these proteins accumulate and aggregate, the proteasome becomes clogged, leading to proteasome inhibition and further accumulation of protein aggregates. Protein aggregation and ER stress trigger pro-apoptotic signaling cascades. Collectively, these factors lead to death of vulnerable neuronal populations.

While the etiology of most neurodegenerative diseases is largely unknown, it is generally recognized that these disorders share a complex set of underlying factors that contribute to disease progression. These include high levels of oxidative and nitrosative stress, excitotoxicity and dysregulation of calcium homeostasis, increased inflammation within the central nervous system (CNS), and significant disruptions in protein homeostasis that ultimately culminate in the death of specific neuronal populations within the brain and spinal cord (reviewed by [1,2,3,4]; Figure 1). It is the death of these neurons and loss of their associated signaling networks that leads to development of the cognitive and motor impairments that are classically associated with diseases such as Alzheimer’s and Parkinson’s disease, respectively.

The failure to develop effective therapeutic agents for neurodegenerative disease may be in large part due to the fact that most current treatments target only single aspects of disease pathology. As such, more recently, current efforts to develop new therapeutic strategies have focused on targeting multiple factors contributing to disease progression. In this regard, polyphenols have garnered significant attention in recent years as pleiotropic agents that are able to modulate many of the features underlying neurodegenerative processes.

Flavonoids are a large class of polyphenolic compounds found in many fruits, vegetables, spices and herbs that are best known for their high levels of intrinsic antioxidant activity. However, in recent years the beneficial effects of flavonoid-rich diets have been found to influence a diverse array of functions, including modulation of inflammatory and apoptotic signaling pathways. Anthocyanins in particular have earned significant attention in this regard, and numerous reports describing their therapeutic benefit for a variety of conditions such as cardiovascular disease and cancer have emerged (reviewed by [5] and [6]).

**Figure 2:**
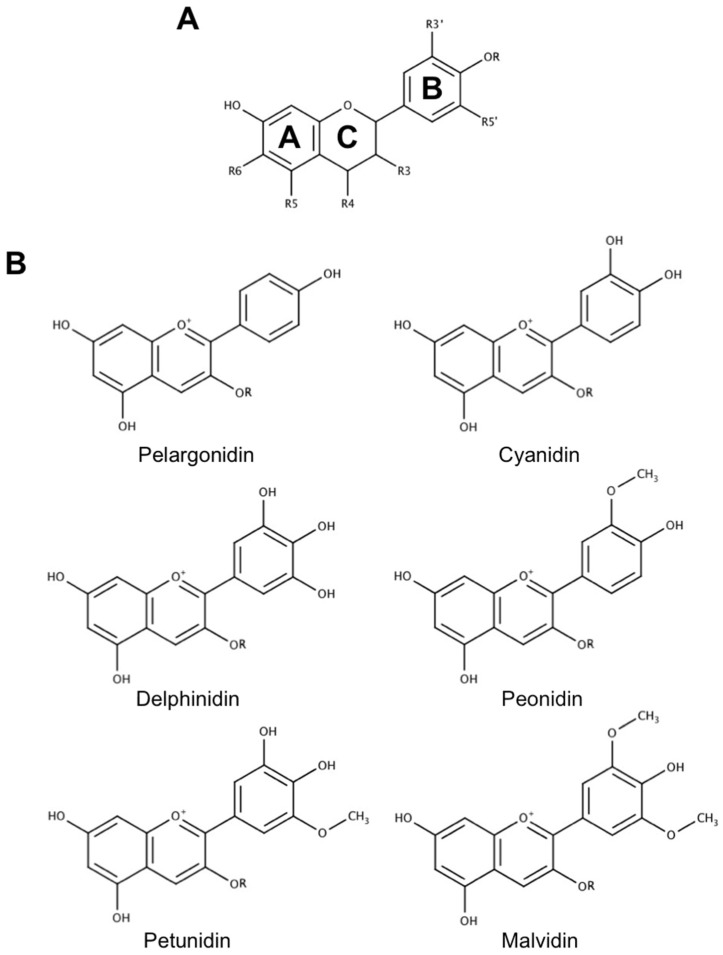
Common anthocyanin structures. (**A**) General flavonoid structure. Flavonoids possess a characteristic three-ring structure that is conserved across all family members. Several classes of flavonoids exist, including anthocyanins, which differ depending on substitutions of the A, B, and C-rings. (**B**) Structures of the six most common anthocyanins. Anthocyanins possess a cationic structure that differs between species predominately in substitutions of the B-ring. Anthocyanins also possess a sugar moiety as a part of their structure, represented as R. Common sugar moieties include but are not limited to glucose, galactose, and rutinose. All structures included in this review were created using MarvinSketch (ChemAxon, Cambridge, MA 02138, USA).

Common anthocyanins are composed of one of six anthocyanidin bases, which differ in molecular structure at the B-ring, and a sugar moiety attached at the third position of the C-ring (Figure 2). These unique cationic compounds are found in high concentrations in a number of fruits, vegetables, and flowers, and are responsible for creating the red, blue, and purple pigmentation observed in many of these species. They are appealing as therapeutic agents for many reasons, one of the most notable being that they are among the most commonly consumed flavonoids in a normal diet and, for this reason, they are generally recognized as safe [7]. This has led to the proposal that anthocyanins may be promising agents for the prevention and treatment of various diseases, including neurodegenerative diseases, that can be easily and safely incorporated into the diet through consumption of anthocyanin-rich foods and beverages or as dietary supplements. Alternatively, the pleiotropic ability of these compounds to modulate multiple aspects of several different diseases fits nicely with research efforts to develop multi-target-directed ligands (MTDLs), an emerging approach to produce new drug candidates for Alzheimer’s disease in particular, and other disorders for which there is a multi-faceted etiology [8]. Common practice in MTDL development is to identify two or more molecules that possess complimentary activities on different disease targets, and then combine structural features of these molecules into a novel drug candidate that possesses the activities of each individual molecule. Since anthocyanins already naturally possess the ability to modulate multiple factors underlying the pathology of several diseases such as cancer and cardiovascular disease, they could act as an appealing scaffold for further efforts to identify and develop effective MTDLs. Indeed, efforts to construct novel MTDLs for Alzheimer’s disease using other flavonoid compounds as a base, particularly flavones and isoflavones, are already underway (reviewed by [9]). Thus, anthocyanins may possess therapeutic potential as both a dietary intervention and as the basis for future drug discovery efforts, making them an interesting class of compounds to consider in the context of developing treatments for neurodegeneration.

However, while anthocyanins have shown great promise for treating many types of disease, it is important to recognize that the bioavailability of these compounds in vivo is relatively low in comparison to their more stable metabolites [10,11]. In particular, phenolic acids derived from anthocyanins are thought to mediate many of the beneficial effects associated with an anthocyanin-rich diet as they are observed to accumulate at high levels in the body [12,13]. Thus, it is of great interest to explore the use of phenolic acid metabolites from anthocyanins in the context of neurodegeneration, and several studies have emerged evaluating the neuroprotective effects of these compounds in the past decade.

In this review, we will summarize the current evidence highlighting the unique activity of anthocyanins and their metabolites against various factors thought to contribute to the development and progression of neurodegenerative diseases. Moreover, we will also explore the current evidence highlighting the use of these compounds in a therapeutic context in various pre-clinical models of neurodegenerative disease and aging. Collectively, this evidence suggests that both anthocyanins and their metabolites possess significant and varied biological activities that make them uniquely suited for further therapeutic development.

## 2. The Use of Anthocyanins as Novel Neuroprotective and Therapeutic Agents in Neurodegenerative Disease

The ability of anthocyanins to attenuate disorders of the CNS is currently under exploration, although data regarding their effects on neurodegeneration are mostly derived from anthocyanin-rich plant extracts. Data regarding the effects of pure anthocyanins are relatively limited, although a few promising studies have been conducted utilizing individual compounds. Nevertheless, several findings have emerged to suggest that anthocyanins ameliorate many of the damaging effects of processes implicated in neurodegeneration such as oxidative and nitrosative stress, excitotoxicity, glial inflammation, protein aggregation, and induction of apoptotic signaling proteins. Furthermore, evidence has accumulated showing that anthocyanins are capable of crossing the blood–brain barrier (BBB), suggesting that these compounds may mediate these effects directly in the CNS where neuronal death takes place [14,15,16,17,18]. Some data have also been reported demonstrating that anthocyanins may be effective therapeutic agents for Parkinson’s disease, Alzheimer’s disease, ALS, and aging. These studies are discussed in detail below.

### 2.1. Absorption and Blood–Brain Barrier Permeability of Anthocyanins

Following ingestion, anthocyanins are swiftly taken up and absorbed into the blood stream where they are transported to target tissues [16,19,20]. The rapid kinetics of anthocyanin absorption into systemic circulation suggests that initial absorption likely takes place in the stomach [21]. This process is thought to be mediated by a bilitranslocase transporter as anthocyanins have been shown to interact with this transporter in several studies [22,23]. A similar mechanism is thought to be responsible for the uptake of these compounds into the CNS as bilitranslocase is present in the endothelial cells that help form the BBB, and it has been shown that anthocyanins are rapidly taken up into vascular endothelial cells in a bilitranslocase-dependent manner [24,25]. Evidence also suggests that flavonoids, such as anthocyanins, may interact with P-glycoprotein transporters, and gain entrance into the brain in this manner [26].

A recent report, in which the anthocyanin cyanidin-3-*O*-glucoside was injected intravenously directly into the blood, suggests that anthocyanin uptake into the brain occurs very rapidly, with the parent anthocyanin being detected within seconds, and anthocyanin derivatives appearing after only minutes [18]. Upon transport into the CNS, anthocyanins have been shown to accumulate in brain tissue at levels up to 0.21 nmol/g of tissue in rodent models [27,28]. One study in older adults also suggests that chronic supplementation with strawberry powder led to accumulation of anthocyanins and their metabolites in the blood, supporting the idea that anthocyanins may persist in tissues over time [13]. Additionally, recent evidence suggests that anthocyanins may undergo rapid methylation and hydroxylation, both in the blood and upon reaching brain tissue, such that one anthocyanin species may be converted to several others (e.g., cyanidin-3-*O*-glucoside may be converted to petunidin-3-*O*-glucoside through methylation) [18]. Accumulation occurs in several tissues, including brain endothelial cells, brain parenchymal tissue, as well as striatum, hippocampus, cerebellum, and cortex [14,15,16]. This finding is of particular interest in that several of these brain regions are known to contain vulnerable neuronal populations whose loss is implicated in several forms of neurodegenerative disease [15].

### 2.2. Antioxidant Effects of Anthocyanins

Oxidative damage is one of the most common features among diverse neurodegenerative diseases, which has made it an appealing therapeutic target. This type of damage occurs when the production of reactive oxygen species (ROS) and reactive nitrogen species (RNS) in the cell overwhelms endogenous antioxidant defenses, resulting in oxidative and nitrosative stress, respectively. Both forms of cellular stress lead to severe oxidative damage of vital cellular macromolecules such as lipid membranes, proteins, and DNA, which culminates in the induction of neuronal cell death [29]. In neurons, the primary sources of oxidative stress are dysfunctional mitochondria, responsible for producing the majority of cellular energy, and functional loss of endogenous antioxidant defenses normally involved in the detoxification of ROS and RNS.

Indeed, under normal conditions, small endogenous antioxidants, such as glutathione (GSH) and Coenzyme Q10 (CoQ10), as well as a number of antioxidant enzymes, such as catalase and superoxide dismutases, are responsible for detoxifying ROS and RNS generated by cellular processes like mitochondrial respiration. However, evidence suggests that in the context of neurodegeneration, the activity of antioxidant enzymes is decreased, and pools of GSH and CoQ10 are depleted [30,31,32]. Loss of intrinsic antioxidant defenses allows ROS and RNS to accumulate and cause oxidative damage to cellular components. In particular, ROS produced at the mitochondria are capable of causing oxidative damage and subsequent mutations in mitochondrial DNA, which encodes major components of mitochondrial respiratory complexes and proteins involved in electron transport, leading to impaired function of the mitochondrial respiratory chain. This in turn results in perturbations in cellular energy, further production of ROS and perpetuation of oxidative stress on a cellular level, ultimately culminating in cell death [33]. Furthermore, mitochondria also play an important role in apoptosis by sequestering cytochrome c, which is tethered to the inner mitochondrial membrane by cardiolipin, and normally acts as an electron shuttle between complexes III and IV in the electron transport chain [34]. It is thought that oxidative damage to cardiolipin leads to a loss of its interaction with cytochrome c, which can then be released into the cytosol where it is able to activate caspase-dependent apoptosis.

Anthocyanins, like many flavonoids, are unique antioxidants in that they are able to scavenge damaging ROS and RNS directly, as evidenced by their high oxygen radical absorption capacity (ORAC) values, in addition to enhancing the cell’s intrinsic antioxidant defenses [35,36,37,38]. Direct scavenging of various ROS including 1,1-diphenyl-2-picrylhydrazyl (DPPH), alkyl, and hydroxyl radicals has been reported using electron spin resonance spectroscopy [39]. Similarly, the major anthocyanin constituents of plum extract have been shown to scavenge superoxide radicals at a higher capacity than other flavonoids such as quercetin [40]. Additionally, one study utilizing sodium nitroprusside (SNP), a nitric oxide-producing agent, has also suggested that some anthocyanins may scavenge nitric oxide radicals and prevent nitrosative stress [41].

The ability of these compounds to directly ameliorate toxic free radicals is thought to mediate some of their neuroprotective effects. Indeed, many studies utilizing anthocyanin-rich fruit extracts, juices, and pure anthocyanins in vitro have demonstrated that these compounds provide potent protection from hydrogen peroxide toxicity in a variety of neuronal cell lines [42,43,44,45,46,47,48,49,50]. Interestingly, similar results were observed in glial cell lines treated with hydrogen peroxide, with anthocyanin or anthocyanidin treatment significantly improving viability and reducing indices of oxidative stress and apoptosis within treated cells [47,51,52]. Additionally, paraquat, another toxin known to cause oxidative stress-induced apoptosis, has been shown to increase intracellular levels of superoxide and hydrogen peroxide, which are ameliorated by treatment with chokecherry concentrate in a neuroblastoma cell line [53]. The anthocyanin, cyanidin-3-*O*-glucoside has also been shown to protect primary cerebellar granule neurons from nitric oxide-induced cell death [41]. As superoxide, hydrogen peroxide, and nitric oxide are the products of many physiological processes, and are known to be elevated in the context of neurodegeneration by processes such as mitochondrial dysfunction and glial inflammation, the ability of anthocyanins to scavenge these radical species suggests that they may be well suited to attenuate oxidative damage in neurodegenerative diseases. This is further supported by an in vivo study in which rats were injected with carbon tetrachloride, an agent that causes generation of free radicals and oxidative damage within several tissues, including brain, after chronic consumption of anthocyanin-rich grape juice [54]. Rats that received grape juice supplementation displayed a significant reduction in markers of oxidative damage such as lipid peroxidation and protein carbonylation compared to rats treated with carbon tetrachloride alone. Anthocyanins from bilberry were also shown to mitigate these markers of oxidative stress induced by conditions of psychological stress in mice that had undergone whisker cutting [55]. Similarly, mice fed a high fat diet, which has been shown to induce significant increases in lipid peroxidation, protein carbonylation, and other markers of oxidative damage in the brain, showed significant improvement in indices of oxidative stress which correlated with enhanced cognitive performance when administered an anthocyanin-rich extract from purple sweet potatoes [56].

**Figure 3:**
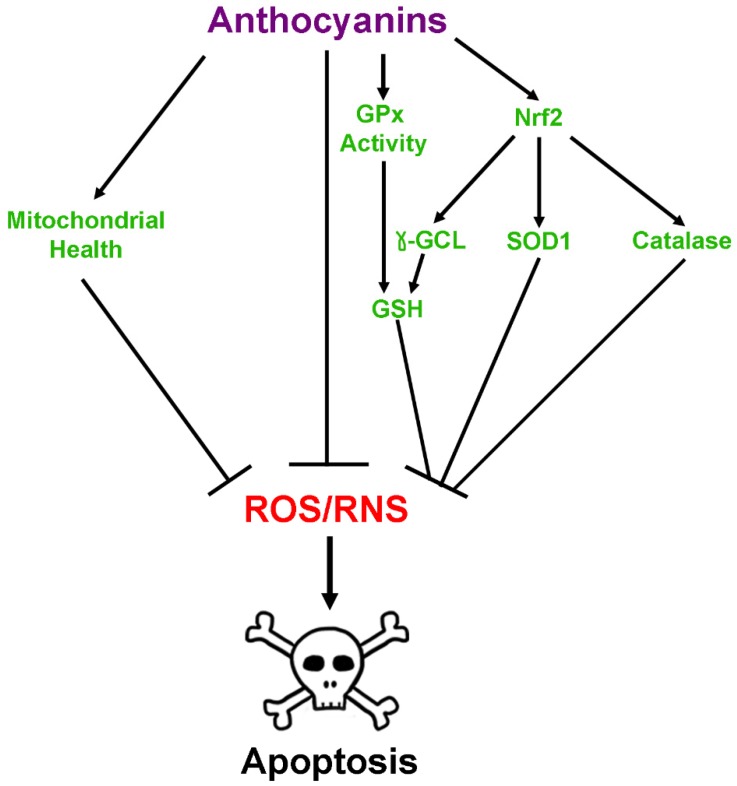
Antioxidant effects of anthoycanins. Anthocyanins modulate damage produced by reactive oxygen and nitrogen species (ROS and RNS) by several mechanisms. These include direct enhancement of glutathione peroxidase (GPx) activity, direct scavenging of ROS and RNS, activation of nuclear factor erythroid 2-related factor 2 (Nrf-2) transcription of antioxidant enzymes, and promotion of mitochondrial health and function. Genes activated by Nrf2 include, but are not limited to, those for catalase, Cu,Zn-superoxide dismutase (SOD1), and gamma-glutamylcysteine ligase (γ-GCL), which increases synthesis of the critical antioxidant, glutathione (GSH). GSH can then be used in conjunction with GPx and another enzyme, glutathione reductase, to scavenge oxidative species. Collectively, these mechanisms detoxify ROS and RNS to prevent apoptosis.

Indirect mitigation of oxidative and nitrosative stress by anthocyanins occurs in several ways and is due primarily to an increase in the levels and activities of antioxidant enzymes. Several reports have documented that treatment with pure anthocyanins or anthocyanin-rich extracts enhances levels of the enzymes catalase, which scavenges hydrogen peroxide, and Cu,Zn-superoxide dismutase (SOD1) both in vitro and in vivo [43,44,45,46,48,49,50,54,57]. Moreover, anthocyanins have been shown to both enhance levels of GSH and prevent its oxidation within neuronal cultures in addition to increasing expression of both glutathione peroxidase and glutathione reductase [53,58,59,60,61]. They have also been demonstrated to directly enhance the activity of glutathione peroxidase, which plays a vital role in detoxifying hydrogen peroxide using GSH [60]. Anthocyanin treatment in glial cells also showed similar results. One study using a glial cell line noted that several different anthocyanin species improved GSH levels following challenge with hydrogen peroxide, while treatment of primary astrocytes with anthocyanin-rich lingonberry extract enhanced thiol levels after treatment with lipopolysaccharide (LPS), indicative of high concentrations of GSH [51,62]. Both pure anthocyanins and anthocyanin-rich extracts have also been shown to reduce mitochondrial oxidative stress and dysfunction induced by Bcl-2 inhibition, hydrogen peroxide, or rotenone toxicity [51,60,61,63]. These activities are thought to be modulated in part by the ability of anthocyanins to induce nuclear factor erythroid 2-related factor 2 (Nrf-2) activity, which acts as a master regulator of many antioxidant genes including catalase and the regulatory subunit of gamma-glutamylcysteine ligase, the enzyme responsible for carrying out the rate limiting step of GSH synthesis, in addition to a host of other phase II detoxification enzymes [35,36,58,64]. This is supported by a recent study demonstrating that aged rats fed an anthocyanin-rich acai pulp diet showed significantly elevated Nrf-2 levels in hippocampus and prefrontal cortex in addition to enhanced levels of antioxidant enzymes such as SOD1 and glutathione S-transferase [57]. Moreover, recent evidence in SH-SY5Y cells suggests that anthocyanins may act directly at the mitochondria to improve mitochondrial redox function, and thereby reduce mitochondrial oxidative stress, by enhancing the activity of complexes I and II, in addition to acting as electron carriers between complex I and subsequent members of the electron transport chain [61]. Anthocyanin-mediated improvements in mitochondrial function have also been observed in SH-SY5Y cells treated with either rotenone, a complex I inhibitor, or a mutant variation of amyloid precursor protein (APP), where they were observed to regulate mitochondrial fission and fusion to preserve healthy mitochondrial dynamics [65]. Collectively, these data suggest that the neuroprotective effects of anthocyanins are mediated through both direct and indirect antioxidant activities within the brain (Figure 3).

### 2.3. Anthocyanins in Calcium Homeostasis and Excitotoxicity

Excitotoxicity is a phenomenon unique to neurons in which excitatory stimuli cause neurons to experience massive calcium influx, membrane depolarization, mitochondrial dysfunction, and subsequent death. This occurs through production of ROS and RNS as well as activation of calcium-dependent pro-death factors, such as calpains [66]. As such, maintaining calcium homeostasis is an essential part of preventing neuronal cell death induced by excitotoxicity. Although the mechanism by which anthocyanins preserve calcium homeostasis is not well understood, several lines of evidence have recently emerged demonstrating that these compounds have significant effects on calcium flux in response to many neurotoxic agents. For example, cells treated with amyloid beta, one of the neurotoxic species thought to underlie the development of Alzheimer’s disease, display significant disturbances in calcium homeostasis leading ultimately to excitotoxicity. However, several studies have reported that anthocyanin treatment in both neuronal cell lines and primary hippocampal cells prevents increases in intracellular calcium caused by this insult [67,68,69]. Anthocyanins have also been shown to promote restoration of calcium levels following depolarization induced by dopamine treatment in primary rat hippocampal neurons [70].

**Figure 4:**
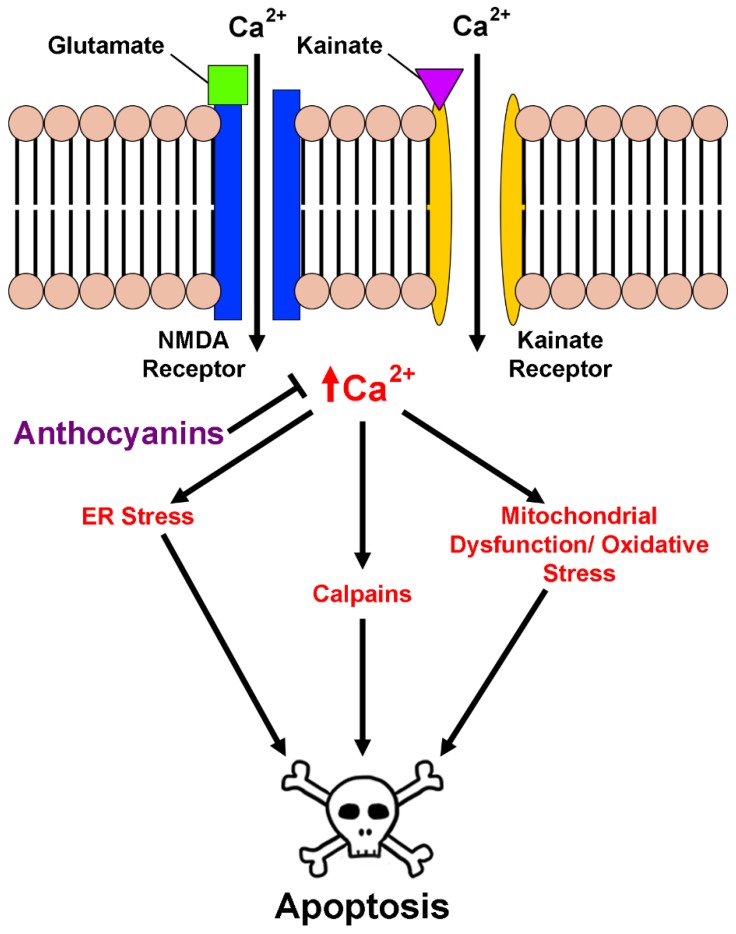
Effects of anthocyanins on calcium homeostasis and excitotoxicity. Binding of the excitatory compounds, glutamate or kainate, to their cognate receptors on the cell membrane causes massive calcium influx into neurons. This calcium influx interferes with the protein folding functions of the ER, resulting in ER stress, activation of the UPR, and subsequent activation of pro-apoptotic signaling cascades if not resolved. Additionally, high levels of intracellular calcium cause membrane depolarization at the mitochondria and uncoupling of the electron transport chain, leading to mitochondrial dysfunction, oxidative stress, opening of the permeability transition pore and release of apoptogenic factors into the cytosol. High calcium concentrations can also lead to direct activation of pro-apoptotic factors such as calpains, leading to cell death. Anthocyanins protect neurons from excitotoxicity by preventing increases in intracellular calcium caused by glutamate and kainate signaling.

The direct effects of anthocyanins on excitotoxic insult have also been described both in vitro and in vivo. In one study, anthocyanins were shown to attenuate increases in intracellular free calcium in both primary hippocampal cells and the HT22 hippocampal cell line in response to treatment with kainic acid, a glutamate receptor agonist [71]. This effect was responsible in part for reducing excitotoxic cell death in these cells. In a similar manner, treatment with the pure anthocyanin, cyanidin-3-*O*-glucoside, inhibited glutamate-induced increases in calcium concentration in rat hippocampal neurons while both this anthocyanin and another, pelargonidin-3-*O*-glucoside, preserved the viability of cerebellar granule neurons in culture [41,72]. Blueberry extract was similarly shown to enhance the viability of primary cortical neuron cultures against glutamate excitotoxicity [73]. It was also recently demonstrated that both anthocyanin-rich black chokecherry extract and the pure anthocyanin, cyanidin-3-*O*-galactoside, are capable of preserving intracellular calcium levels when co-administered with glutamate in HT22 cells, resulting in preserved cellular viability and mitochondrial function [59]. These effects have also been observed in vivo using a model of retinal ganglion cell degeneration. Intraocular injection of mice with *N*-methyl-d-aspartate (NMDA) induced excitotoxicity by stimulating calcium influx through ionotropic glutamate receptors, causing wide-spread cell death in injected mice; however, simultaneous injection with anthocyanin-rich bilberry extract dramatically attenuated these effects [74]. Comparable results were observed in rats fed a blueberry-enriched diet, which contains high levels of anthocyanins, that received hippocampal injections of kainic acid. Indeed, blueberry supplementation significantly enhanced neuronal survival in the CA1 region of the hippocampus and attenuated kainic acid-induced cognitive deficits [75]. The effects of anthocyanins on excitotoxicity and calcium regulation are summarized in Figure 4.

### 2.4. Anti-Neuroinflammatory Activity of Anthocyanins

The anti-inflammatory effects of anthocyanins are well established in non-neuronal systems, particularly those related to cardiovascular disease (reviewed by [5]). However, their role in reducing neuroinflammation is less understood and the topic of current exploration. In the brain, inflammatory responses are mediated by microglia, the resident immune cells of the CNS, which are capable of secreting a host of neurotoxic factors when exposed to aversive stimuli. While acute inflammatory responses are beneficial for ridding the brain of dying cells or foreign pathogens, prolonged or chronic inflammation, which has been implicated as a factor underlying neurodegeneration in several diseases, can lead to the death of surrounding neurons [76].

Several recent studies have assessed the anti-inflammatory effects of anthocyanins and anthocyanin-rich extracts on the BV2 and C8-B4 mouse microglial cell lines. These studies have demonstrated that anthocyanin treatment is capable of significantly reducing induction of pro-inflammatory proteins such as inducible nitric oxide synthase (iNOS) and cyclooxygenase-2 (COX-2) in response to stimulation with lipopolysaccharide (LPS), a component of bacterial cell walls that is known to induce a pronounced inflammatory response. Furthermore, these studies demonstrated that treatment with anthocyanin-rich extracts significantly attenuates production and secretion of nitric oxide, interleukin-1β (IL-1β) and tumor necrosis factor-α (TNF-α) [47,77,78,79,80,81,82,83]. Similar results have been observed in HAPI cells, a rat microglial cell line, in which it was shown that pretreatment with tart cherry extract significantly reduced expression of COX-2 in cells challenged with LPS, although expression of iNOS was not significantly affected [84]. Despite this finding, it was noted that nitric oxide production was significantly attenuated, which may suggest a direct effect of tart cherry treatment on NOS activity. Additionally, inflammatory markers such as TNF-α were also significantly reduced in this paradigm [84]. Modulation of pro-inflammatory signaling pathways is also reported as levels of active c-Jun-N-terminal kinase (JNK), p38-mitogen activated protein kinase (p38-MAPK), extracellular signal-regulated kinase 1/2 (ERK1/2), and Akt, are all significantly reduced [78,79,80,82]. Reduction in the activity of these signaling pathways correlates with reductions in activated nuclear factor-κB (NF-κB), and prevention of its translocation to the nucleus, where it is capable of mediating the transcription of many pro-inflammatory genes [77,78,79,80,81,82,83]. It was also recently observed that the anthocyaninidin, cyanidin, modulates inflammatory responses through decreasing expression of toll-like receptor 4 (TLR4), which lies upstream of NF-κB activation, in neuroblastoma cells treated with amyloid beta protein. This led to concomitant decreases in NF-κB nuclear translocation, iNOS expression, and nitric oxide production [64]. Likewise, in primary microglia, blueberry extract, which is rich in anthocyanins, was shown to promote phagocytosis of amyloid beta peptides and to reduce microglial inflammation in a manner that was dependent on ERK1/2 inhibition [85]. Moreover, it has been shown that conditioned media collected from BV-2 microglial cells treated with LPS and anthocyanins are less toxic to the HT22 neuronal cell line in culture, indicating that reductions in microglial inflammation promote neuronal survival [77].

Interestingly, similar findings have been made in primary astrocytes. While studies of neuroinflammation have focused predominately on microglia as the major contributors to inflammation in the CNS, reactive astrocytes are capable of secreting many of the same inflammatory factors as microglia, and recent evidence suggests that treatment of primary astrocytes with either anthocyanin-rich lingonberry extract or cyanidin significantly attenuates the inflammatory response of astrocytes to LPS [62,86]. Indeed, this research demonstrated that lingonberry extract improved cellular viability, and reduced several markers of inflammation and oxidative stress, such as pro-inflammatory cytokine and nitric oxide secretion, and accumulation of ROS, which may help astrocytes maintain a quiescent state [62,86]. Moreover, lingonberry extract blunted the activity of acetylcholinesterase, which may play an important role in inhibiting induction the NF-κB pathway in glial cells in addition to enhancing cholinergic signaling, which has been shown to relieve symptoms of Alzheimer’s disease [62]. Though few in number, these studies clearly demonstrate that treatment with anthocyanins or anthocyanin-rich extracts is capable of ameliorating many of the inflammatory effects of microglial and astrocytic activation.

**Figure 5:**
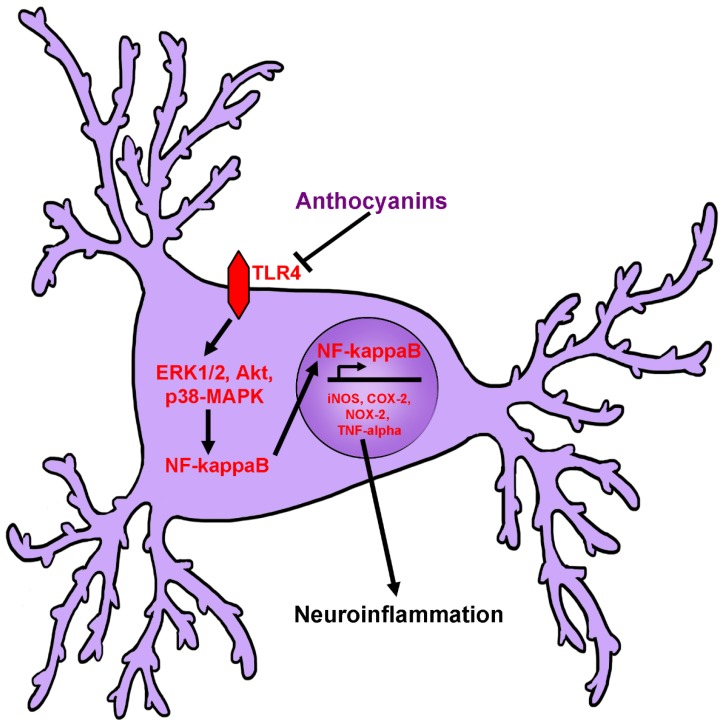
Effects of anthocyanins on neuroinflammation. Inflammatory stimuli, such as deposits of aggregated proteins, cause activation of toll-like receptor-4 (TLR4), and downstream induction of extracellular regulated signal kinase 1/2 (ERK1/2), Akt, and p38-mitogen-activated protein kinase (p38-MAPK), which subsequently activate nuclear factor-κB (NF-κB) in microglia and astrocytes. NF-κB then translocates to the nucleus and initiates transcription of pro-inflammatory genes including, but not limited to inducible nitric oxide synthase (iNOS), cyclooxygenase-2 (COX-2) NADPH-oxidase-2 (NOX-2), and tumor necrosis factor-α (TNF-α). It is thought that anthocyanins inhibit this pathway by blocking activation of TLR4, ERK1/2, Akt, and p38-MAPK.

These findings are mirrored in vivo in several experiments demonstrating that mice or rats receiving anthocyanins displayed a marked decrease in neuroinflammatory markers and increases in anti-inflammatory markers following injection with LPS [77,87,88,89]. For example, levels of both iNOS and COX-2 were significantly reduced in the brains of mice treated with anthocyanins from purple sweet potato after LPS treatment, correlating with significant improvements in cognition and memory tasks [87]. In the same model, it has been reported that anthocyanins from black soy bean and Korean black beans decrease indices of oxidative stress, and several markers of inflammation including reactive microglia, NF-κB expression, JNK phosphorylation, and production of pro-inflammatory cytokines like TNF-α [77,88]. These observations correlated with significant decreases in pro-apoptotic proteins, increases in pro-survival signaling and enhanced neuronal survival [77,88]. Additionally, black bean anthocyanins promoted expression of synaptic signaling proteins and improved cognitive deficits in LPS-treated mice [77]. Similarly, mice treated with LPS and administered anthocyanins from bilberry also show improvements in inflammatory markers in addition to displaying enhanced expression of anti-inflammatory cytokines, such as IL-4, and improved cognition [89]. Collectively, these in vivo studies confirm the anti-inflammatory effects of anthocyanins against LPS that have been reported in vitro. Purple sweet potato extract also had a significant anti-inflammatory effect in mice receiving a high fat diet, where it was observed to prevent nuclear translocation of NF-κB and activation of MAPK pathways, reduce expression of iNOS and COX-2, and decrease expression of several pro-inflammatory cytokines [90]. Similarly, anthocyanins extracted from black goji berry were shown to be protective in a model of ischemic stroke where they were seen to significantly reduce expression of inflammatory cytokines, NF-κB, and NLRP3, part of the inflammasome pathway that is responsible for generating pro-inflammatory cytokines such as IL-1β [91]. Blueberry extract was also shown to decrease inflammation associated with excitotoxic insult with kainic acid in rat hippocampus in a comparable manner, which corresponded with improved learning and memory [92]. Considered together, these studies indicate that anthocyanins may effectively attenuate inflammation in glial cells, and thus, reduce damage in surrounding neurons (Figure 5).

### 2.5. Anthocyanins and Regulation of Protein Homeostasis

Though limited, new data are emerging to suggest that anthocyanins may also exert neuroprotective effects by directly preventing protein aggregation and by stimulating autophagy. As protein aggregation has been suggested to play a significant role in neuronal death for many diseases, the ability of therapeutic agents to inhibit protein oligomerization into toxic plaques and fibrils is desirable. Indeed, protein aggregates are a hallmark of Alzheimer’s disease, in which amyloid beta peptides form toxic plaques, and hyperphosphorylated tau proteins accumulate into neurofibrillary tangles, Parkinson’s disease, in which α-synuclein is seen to form characteristic Lewy bodies, and ALS in which mutant and oxidized forms of various proteins, particularly SOD1 and TAR-DNA-binding protein-43 (TDP-43) among others, have been shown to forms large aggregates throughout the cell (Table 1).

The pure anthocyanin cyanidin-*3-O*-glucopyranoside has been shown to directly interfere with oligomerization of amyloid beta peptides, one of the major constituents of senile plaques observed predominately in Alzheimer’s disease [105]. Similarly, the aglycon, malvidin, and its glucoside conjugate have also been reported to potently inhibit amyloid beta oligomerization into toxic fibrils, as have anthocyanin-rich extracts derived from bilberry, which was also reported to reduce the toxicity of such aggregates, and several other berry fruits [47,85,106,107]. Cyanidin-3-*O*-glucopyranoside has shown similar activity, in addition to inhibiting the interaction of amyloid beta with cellular membranes [108]. These findings were further corroborated by another study demonstrating that a unique blend of pure anthocyanins and anthocyanidins prevented amyloid beta oligomerization directly in addition to preventing amyloid beta-induced tau phosphorylation in a neuronal cell line, which may help prevent tau protein aggregate formation in Alzheimer’s disease [42]. Though the mechanism by which anthocyanins inhibit aggregate formation is currently unknown, the ability of these compounds to disrupt the formation of toxic oligomers is promising for their therapeutic efficacy. Further data is needed to determine if anthocyanins are also able to disrupt toxic aggregate formation of other protein species, such as SOD1 in ALS and α-synuclein in Parkinson’s disease.

Recent studies have also reported the ability of anthocyanin-rich extracts to modulate autophagy. This process, in addition to proteasomal degradation, is vital for clearing toxic aggregates and misfolded proteins from the intracellular space to prevent neuronal death [56,57,70]. Acai fruit extract has been shown to be beneficial for stimulating autophagy both in vitro and in vivo [57,70]. Indeed, treatment of HT22 cells with the autophagy inhibitors wortmanin and bafilomycin A1 caused significant accumulation of poly-ubiquitinated proteins, which was corrected by treatment with acai pulp extracts [70]. Additionally, these extracts significantly enhanced turnover of autophagosomes and enhanced activation of mammalian target of rapamycin (mTOR), one of several regulators of the autophagy pathway. These results were confirmed in vivo in the brains of aged rats treated with acai pulp extracts, demonstrating up-regulation of autophagy markers, such as mTOR activation [57]. Tart cherry supplementation in aged rats showed comparable results, enhancing markers of autophagy in the hippocampus of treated animals [109]. Similarly, a more recent study demonstrated that extract from purple sweet potatoes significantly up-regulated markers of autophagy in the hippocampus of mice fed a high fat diet, and demonstrated that this process was dependent on activation of AMP-activated protein kinase (AMPK) [56]. These changes correlated with a significant reduction in neuronal apoptosis within the hippocampus and significant increases in brain-derived neurotrophic factor (BDNF) levels, which are reduced in the context of autophagic impairment. [56]. Taken together, these results suggest that anthocyanins and anthocyanin-rich extracts may modulate processes such as protein aggregation and autophagy to correct disruptions in protein homeostasis observed in neurodegenerative disease, though further data is needed to confirm this hypothesis.

**Figure 6:**
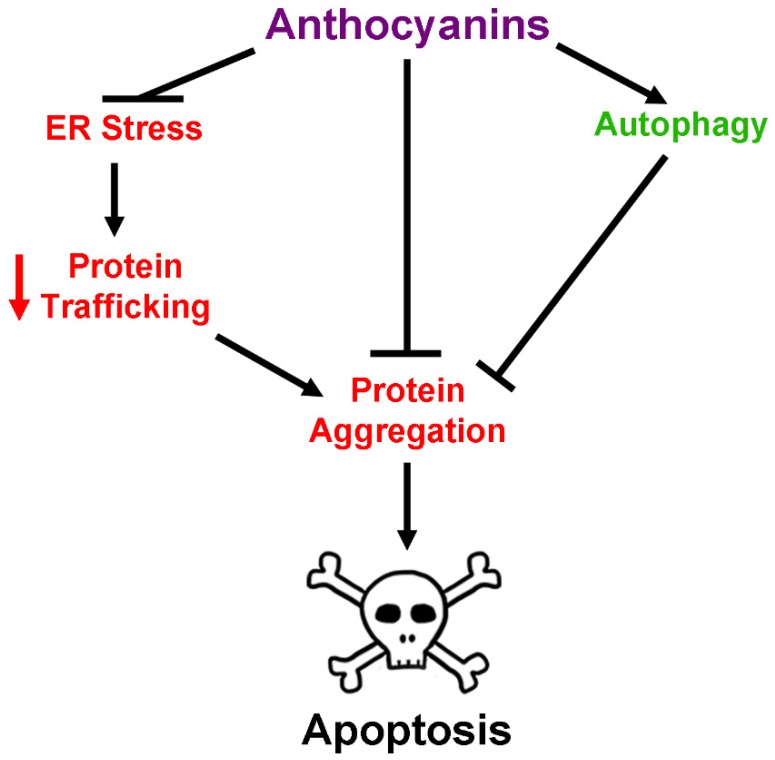
Effects of anthocyanins on protein dysregulation and homeostasis. Protein homeostasis is disrupted in several ways in neurodegenerative disease. Increased levels of ER stress cause significant decreases in protein trafficking to other organelles such as the Golgi apparatus. Decreased protein trafficking results in accumulation of misfolded and mutant proteins, causing protein aggregates to form. Formation of these toxic aggregates then contributes to induction of neuronal apoptosis. Anthocyanins modulate this process by reducing ER stress, directly inhibiting the formation of toxic protein aggregates, and stimulating autophagy processes to clear aggregates formed within the neuron.

ER stress is another major consequence of protein misfolding and aggregation in neurodegeneration, and results in induction of the unfolded protein response (UPR). If protein aggregation and misfolding cannot be resolved by the UPR, ER stress-induced apoptosis may occur, resulting in neuronal loss, a phenomenon that has been shown in several neurodegenerative diseases [110]. Studies assessing the effect of anthocyanins on ER stress are currently limited, but evidence has begun to accumulate demonstrating their efficacy for mitigating this aspect of neurodegeneration. In a recent report using a neuroblastoma cell line, it was demonstrated that cyanidin significantly attenuated cell death induced by amyloid beta by decreasing expression of ER-stress and unfolded protein response markers such as protein kinase RNA-like endoplasmic reticulum kinase (PERK), C/EBP homologous protein (CHOP), and activating transcription factor 6 (ATF6), and preventing activation of caspase-12 and calpain, executioners of ER stress-induced apoptosis [111]. Moreover, treatment of a photoreceptor cell line exposed to damaging blue light with bilberry extract or its pure constituent anthocyanins prevented protein aggregation and poly-ubiquitination, in addition to attenuating increases in markers of the unfolded protein response, such as bip and grp94 [112]. There is also some evidence to suggest that anthocyanins may regulate ER stress in vivo, thereby preventing ER stress-induced apoptosis. In a mouse model of cognitive impairment induced by domoic acid, mice experienced a significant increase in levels of ER stress markers such as PERK and apoptosis signal-regulating kinase 1 (ASK1) in hippocampal tissue [113]. However, treatment with purple sweet potato extract entirely mitigated this effect, returning these markers to normal levels and preventing ER stress-induced apoptosis. It has also been shown that anthocyanin-rich bilberry extract up-regulates chaperone proteins associated with inhibition of ER stress-induced apoptosis in a mouse model of optic nerve-crush injury, preventing the death of retinal ganglion cells [114]. Though to our knowledge, these are the only studies to date exploring the effects of anthocyanin extracts on neuronal ER stress in vivo, they demonstrate that these compounds may show some benefit in neurodegenerative disorders for which ER stress and concomitant protein dysregulation is an underlying factor (Figure 6).

### 2.6. Anti-Apoptotic Effects of Anthocyanins

Induction of neuronal cell death pathways is the ultimate culmination of pathological events associated with neurodegeneration. Anthocyanins are known to modulate several signaling pathways involved in cell death and survival. Their effects on apoptosis specifically are well documented and have been shown to occur in both caspase-dependent and caspase-independent manners [115]. For example, in both an in vitro study in a neuronal cell line and a d-galactose mouse model of aging, treatment with purple sweet potato extract suppressed activation of pro-apoptotic proteins such as JNK while also preventing mitochondrial release of cytochrome c and subsequent execution of apoptotic signaling pathways [69,116]. These effects were mediated by activation of phosphoinositide-3-kinase (PI3K), which is an upstream activator of Akt, a major regulator of pro-survival signaling [116]. In agreement with these findings, it was also reported that anthocyanins and anthocyanin-rich extracts attenuate p53 and JNK-dependent apoptosis and reduce caspase-3 activity in a model of ischemic injury induced by cerebral artery occlusion, which shares many pathological features with neurodegenerative disease such as oxidative stress, excitotoxicity, neuroinflammation, and ultimately neuronal death [117,118].

**Figure 7:**
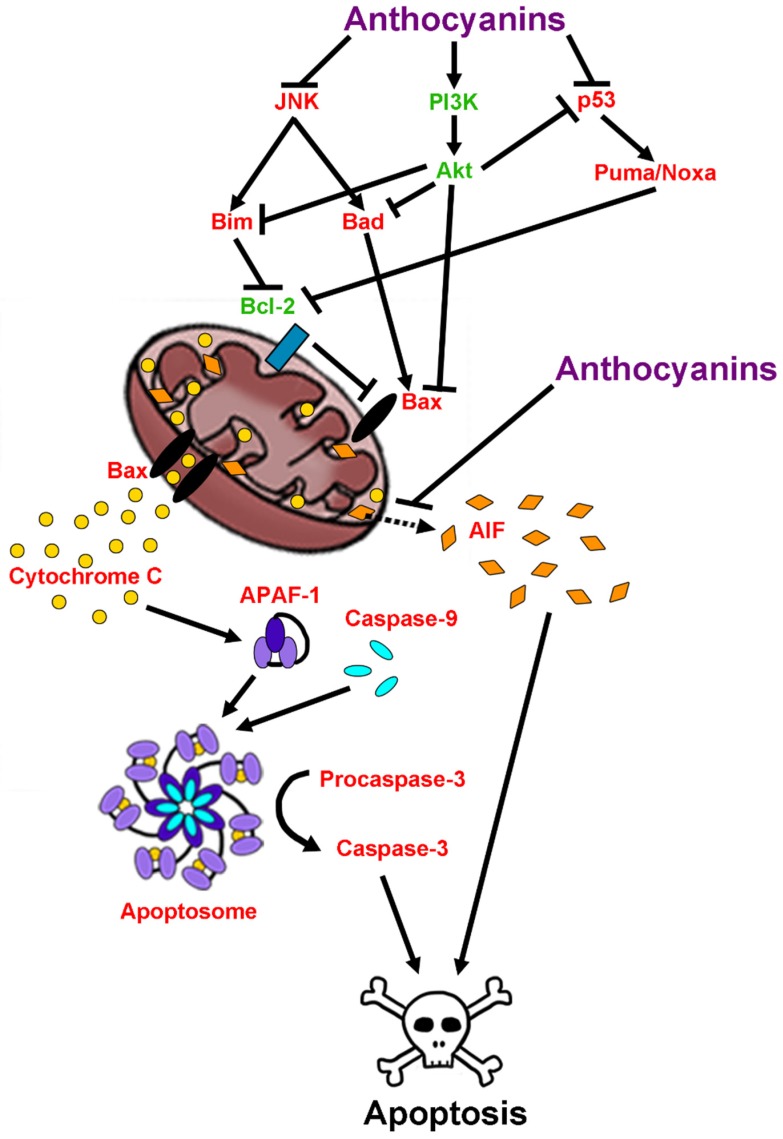
Effects of anthocyanins on pro-survival and pro-apoptotic signaling pathways. Anthocyanins modulate several signaling pathways involved in cell survival and death. Anthocyanins inhibit the activity of c-Jun N-terminal kinase (JNK) and p53, which are responsible for activating pro-apoptotic family members of the Bcl-2 family of proteins, Bim, Bad, Puma, and Noxa. Bim, Puma, and Noxa are known to inhibit the pro-survival functions of B-cell lymphoma-2 (Bcl-2), causing activation of the pro-apoptotic protein, Bax. Bax can also be activated by interaction with Bad. Bax then forms pores in the mitochondrial membrane, allowing the release of cytochrome c from mitochondria. Cytochrome c interacts with apoptosis protease activating factor-1 (APAF-1) and caspase-9 to form the apoptosome. The apoptosome then cleaves procaspase-3 to form active caspase-3, stimulating apoptosis. Anthocyanins also enhance the activity of the phosphoinositide-3-kinase (PI3K)/Akt pro-survival signaling pathway, which inhibits activity of pro-apoptotic Bcl-2 family members including Bim, Bad, and Bax, in addition to inhibiting the activity of p53. This activity inhibits entry of neurons into caspase-dependent apoptosis. Alternatively, anthocyanins have also been shown to inhibit caspase-independent apoptosis by blocking the translocation of apoptosis inducing factor (AIF) from mitochondria to the cytosol and subsequently, the nucleus (dashed arrow).

There are also reports that anthocyanins are capable of modulating the expression of Bcl-2 family members. Indeed, in a cellular model of 6-hydroxydopamine (6-OHDA) toxicity, a chemical often used to induce a Parkinsonian phenotype in mice and rats, treatment with an anthocyanin-rich extract from mulberry elevated expression of pro-survival Bcl-2 protein while suppressing expression of the pro-apoptotic Bax protein [119]. Similar results were observed in SH-SY5Y cells treated with the aglycon, cyanidin, and 1-methyl-4-phenylpyridinium (MPP^+^), which acts as a mitochondrial complex I inhibitor to induce neurotoxicity [120,121]. In this model, cyanidin treatment dose-dependently prevented cell death, and lowered the ratio of Bax:Bcl-2 ratio [120]. This has been further validated in vivo in a study demonstrating that anthocyanin treatment significantly reduced both the Bad:Bcl-xL ratio and the Bax:Bcl-2 ratio, and increased cellular survival in a rat model of ischemic stroke [91]. Finally, anthocyanins have also demonstrated the ability to prevent release of apoptosis inducing factor (AIF) from mitochondria, likely accounting for the ability of these compounds to effectively mitigate caspase-independent apoptosis [122]. In sum, these data indicate that anthocyanins are not only capable of ameliorating many factors implicated in causing neuronal death in neurodegenerative disease, but also pro-apoptotic signaling itself, thus targeting disease progression at multiple levels (Figure 7).

### 2.7. Anthocyanins as Therapeutic Agents in Aging and Neurodegenerative Disease

Since anthocyanins display impressive pleiotropic effects, combating multiple facets of the neurodegenerative process, it has been recently hypothesized that they may be effective therapeutic agents for the treatment of neurodegenerative diseases. Indeed, several reports have indicated that anthocyanins and anthocyanin-enriched extracts are capable of relieving the motor and cognitive deficits associated with Parkinson’s disease, Alzheimer’s disease, and ALS.

Parkinson’s disease is often modeled chemically using injection of the toxin 1-methyl-4-phenyl-1,2,3,6-tetrahydropyridine (MPTP), which has been shown to induce the death of nigrostriatal dopaminergic neurons, the major neuronal population affected in Parkinson’s disease. In mice that were treated with mulberry extract before MPTP injection, however, this effect was significantly reduced and dopaminergic neurons were preserved [119]. This observation correlated with decreases in pro-apoptotic protein expression and significant improvements in Parkinsonian symptoms. Additionally, a more recent study in the same model further reported that treatment with mulberry extract also significantly attenuated increases in expression of both alpha-synuclein and ubiquitin, the primary constituents of Lewy bodies, caused by MPTP treatment [123]. A similar effect was described in another chemical model of Parkinson’s disease using rats injected with 6-OHDA. Studies using 6-OHDA have demonstrated that this compound causes significant oxidative damage and neuronal death in relevant neuronal populations associated with Parkinson’s disease [124]. Rats injected unilaterally with 6-OHDA showed decreased numbers of dopaminergic neurons within the lesioned *substantia nigra* and elevation in levels of lipid peroxidation [125]. These effects were essentially ablated, however, by administration of the aglycon anthocyanin derivative, pelargonidin, which corresponded to significant improvements in motor function. A diet rich in blueberries also had beneficial effects in this model, as animals administered this diet displayed a transient increase in reactive microglia that had resolved one month post-lesion, which correlated with recovery of dopaminergic neurons as indicated by tryosine hydroxylase immunoreactivity in striatal tissue [126].

Positive findings have also been shown for the treatment of Alzheimer’s disease and age-related cognitive impairment with anthocyanin-rich extracts. Several studies have emerged indicating that anthocyanins and anthocyanin extracts attenuate many of the aspects associated with amyloid beta toxicity in vitro, such as decreases in cellular viability, increases in ROS and intracellular calcium, beta-secretase expression, down-regulation of pro-survival proteins, and elevation of pro-apoptotic signaling proteins [42,64,67,68,69,108,111,127,128,129,130]. Similar findings have been made in the BV-2 microglial cell line, where it has also been demonstrated that anthocyanin treatment significantly reduces markers of inflammation such as NF-κB, iNOS, COX-2 and TNF-α expression, and JNK activation among others [131].

In addition to in vitro evidence, several reports have emerged assessing the efficacy of anthocyanins in various animal models of Alzheimer’s disease. A study in the APP/PS1 mutant mouse model of Alzheimer’s disease, which expresses the transgenes for both mutant APP and mutant presenilin-1 (PS1), demonstrated that transgenic mice develop significant cognitive impairments in spatial working memory and accumulation of amyloid beta in brain tissue [132]. This accumulation was significantly reduced by treatment with anthocyanins isolated from bilberry and black currant, and both extracts were able to prevent cognitive decline and improve behavioral abnormalities. These findings were recently corroborated in another study, which also indicated that the positive effects on cognition observed with bilberry extract supplementation were not due to decreased plaque load, but rather were attributed to an increase in plaques containing a less neurotoxic form of amyloid beta [106]. In the same model, another study demonstrated beneficial effects on cognition in APP/PS1 mice treated with the pure anthocyanin, cyanidin-3-*O*-glucopyranoside that correlated with activation of peroxisome proliferator activated protein-γ (PPAR-γ), which has been shown to play a role in amyloid beta processing and inflammation [108]. Korean black bean anthocyanins have also shown positive effects in this model, where they have been shown to stimulate the PI3K/Akt signaling pathway and up-regulate Nrf2 antioxidant signaling to enhance neuronal survival and preserve synaptic function [129]. These alterations corresponded with enhanced cognitive function. Another study in a similar mouse model, which expresses the Swedish variant of mutant APP (APPsw), demonstrated that anthocyanin-rich extract from pomegranate also decreases levels of amyloid beta protein in addition to reducing levels of pro-inflammatory cytokines such as TNF-α, IL-1β, and IL-6 in vivo [133]. Likewise, a study by Qin et al. [134] demonstrated that treatment with cyanidin-3-*O*-glucoside prevented cognitive impairment induced by amyloid beta intracerebroventricular injection. Positive findings were also reported by Badshah et al. [67] and Kim [130] in anthocyanin-treated rats injected with amyloid beta, which showed significant reductions in markers of apoptosis and Alzheimer’s disease in cortical and hippocampal tissue. More recent reports have confirmed these data, showing that anthocyanins in both mice and rats treated with amyloid beta significantly improved cognitive deficits induced by amyloid beta injection and improved several indices of both oxidative stress and neuroinflammation [131,135,136]. These findings are in good agreement with a study conducted in senescence accelerated mouse prone 8 (SAMP8) mice, which are used as a model of accelerated aging. These mice experience significant cognitive impairment, which correlates with deposition of amyloid beta plaques, reminiscent of those observed in Alzheimer’s disease. Additionally, these mice display elevated levels of stress kinase signaling in the brain mediated by JNK and p38-MAPK. Treatment with an anthocyanin-rich extract from mulberry preserved cognitive function and significantly reduced amyloid plaque burden and stress kinase signaling in the brains of treated mice [137]. Anthocyanins have also been shown to have positive effects in a model of streptozotocin-induced dementia, which has been used recently as a model of sporadic Alzheimer’s disease. In this context, anthocyanins were shown to enhance antioxidant status within the brain and reduce the activity of acetylcholinesterase to improve behavioral performance in cognitive tests [138,139].

Human studies in older adults at risk for dementia have also yielded promising results with regards to anthocyanin consumption. The dietary introduction of blueberries and Concord grape juice, which are rich in anthocyanins, significantly improved mild memory impairment in treated individuals [140,141,142]. Moreover, a recent study also demonstrated that a 12-week dietary intervention with anthocyanin-rich cherry juice significantly improved short and long-term memory in elderly adults with mild to moderate dementia [143].

In addition to beneficial effects in Parkinson’s disease and Alzheimer’s disease models, anthocyanins have also been shown to have some efficacy for the treatment of ALS. An anthocyanin-enriched extract from strawberries was shown to significantly delay disease onset and extend survival in the G93A mutant SOD1 mouse model of ALS when administered prior to symptom development [144]. These alterations in disease course were accompanied by preservation of motor function and muscle strength, which correlated with reductions in reactive astrocytosis, and preservation of both motor neurons in lumbar spinal cord and neuromuscular junctions in muscle tissue [144]. However, as this is the only study to date assessing the effects of anthocyanins in this disease, further exploration is needed to verify these observations.

A growing body of evidence has also emerged indicating that anthocyanin supplementation may positively impact aging in rodent models and healthy older adults. Several studies utilizing various anthocyanin-rich extracts have been conducted in the d-galactose model of aging, and have demonstrated that anthocyanin treatment significantly attenuates numerous deleterious factors contributing to aging [116,145,146,147,148,149,150,151]. Studies with purple sweet potato extract have shown that administration to d-galactose-treated mice significantly improves cognitive behavior and that this improvement correlates with preservation of proteins involved in both pre-and post-synaptic function, activation of pro-survival and antioxidant signaling pathways, such as PI3K and SOD1 respectively, and suppression of pro-apoptotic and pro-inflammatory protein expression, such as JNK, and NF-κB [116,145,146]. Similar results were reported in rats using anthocyanins extracted from black soybean [149,151]. A blueberry-enriched diet also improved outcomes in rats treated with d-galactose by promoting expression of Bcl-2 and antioxidant enzymes, and reducing the expression of Bax and acetylcholinesterase activity, the latter of which is consistent with the anti-inflammatory activity of anthocyanins in glial cells, and their ability to modulate synaptic transmission in neurons [148]. Enhanced expression of antioxidant enzymes and decreases in markers of oxidative stress in the brains of d-galactose treated mice were also reported after administration anthocyanins extracted from black rice [147]. Interestingly, this study also observed that black rice anthocyanins were able to reduce expression and activity of monoamine oxidase-B, which is important for preserving levels of monoamine neurotransmitters, such as dopamine, and has been shown to be therapeutic for Parkinson’s disease [147]. These results were recently corroborated in another study using the d-galactose model of aging and anthocyanins extracted from black chokecherry, in which improvements in cognitive behavior correlated with elevations in the levels of several monoamines and antioxidant enzymes, as well as decreases in pro-inflammatory proteins and markers of DNA damage and apoptosis, such as p53 [150]. Lastly, two studies using extract isolated from black soybeans and black goji berries in rats treated with d-galactose also demonstrated the significant anti-inflammatory and anti-apoptotic effects of anthocyanins, while also highlighting the ability of anthocyanins to significantly reduce beta-secretase expression, which is responsible for the generation of toxic amyloid beta peptides, and expression of amyloid beta itself [149,151]. Moreover, these studies illustrated that anthocyanin treatment was capable of reducing receptor for advanced glycation end products (RAGE) protein expression, which acts as a receptor for advanced glycation end products that accumulate with age and cause significant dysfunction within the brain [149,151]. As other studies have shown that anthocyanins are also capable of decreasing production of advanced glycation end products themselves in vitro (see [47,152]), this pathway may represent another therapeutic target for anthocyanin treatment in aging and neurodegeneration.

The effects of both pure anthocyanins and anthocyanin-rich extracts on the deleterious aspects of chronological aging have also been assessed in numerous studies [15,17,57,81,109,153,154,155,156,157,158,159,160,161,162]. Concord grape juice or a diet enriched in blackberry, for example, were shown to significantly attenuate age-related declines in cognitive and motor performance in old rats [158,159]. Similar behavioral findings were achieved in aged rats by supplementing their diet with either freeze-dried strawberry or blueberry powder, which was also observed to ameliorate ROS and age-related declines in insulin-like growth factor-1, and enhance hippocampal neurogenesis [157,161]. Tart cherry supplementation has also been evaluated in aged rats, and while no improvements on motor performance were observed following administration, dietary intervention with tart cherry enhanced working memory [109]. Interestingly, it was also shown that aged rats receiving acai pulp displayed improvements in cognition and that serum collected from these rats, when applied to BV-2 microglial cells, caused the microglia to produce less inflammatory markers, such as iNOS and TNF-α, than cells treated with serum from animals fed a control diet [154]. Both tart cherry and acai pulp were demonstrated to modulate several processes involved in aging, reversing declines in antioxidant status, decreasing inflammation through reductions in COX-2, NOX-2 and NF-κB expression, and improving deficits in autophagy within the frontal cortex and hippocampus of aged animals [57,109]. Blueberry supplementation is perhaps the most well-studied dietary intervention in aging animals, and several studies have demonstrated that both short and long-term supplementation with freeze-dried blueberry powders significantly improves cognition in aged rats [15,17,81,153,155,156,157,160,161]. Intriguingly, improvements in working memory in aged animals receiving blueberry supplementation correlated with decreased NF-κB expression and enhanced ERK and cAMP response element binding protein (CREB) activity, and elevated levels of brain-derived neurotrophic factor (BDNF) in hippocampus, a pathway thought to be important for memory formation [17,81]. This finding was confirmed in a more recent study in which both blueberry powder and pure anthocyanins incorporated into the diet of aged rats significantly enhanced both BDNF protein levels in the hippocampus and spatial working memory [156]. A blueberry enriched diet has also been shown to enhance long-term potentiation in the hippocampus of aged rats by enhancing activity of NMDA receptors, suggesting that intervention with anthocyanin-rich extracts, particularly those from blueberry, may influence multiple processes involved in learning and memory to improve cognition in the context of aging [162]. In addition to findings in animal models of aging, recent clinical studies have also indicated that dietary supplementation with freeze-dried blueberry powder or blueberry extracts also has a positive, albeit modest, impact on memory in healthy older adults [163,164,165]. Moreover, supplementation with blueberry concentrate was shown to enhance activity in regions of the brain associated with cognitive tasks as they were being performed [163].

## 3. The Use of Anthocyanin Metabolites as Novel Neuroprotective and Therapeutic Agents in Neurodegenerative Disease

While anthocyanins have proven to be promising therapeutic candidates in a preclinical context, it is important to note that the overall bioavailability of parent anthocyanins is very low despite their high degree of bioactivity. Indeed, the concentrations that are needed to achieve neuroprotection in cultured neurons far exceed physiological concentrations of anthocyanins observed in brain tissue from supplemented animals [166,167]. Furthermore, several reports have indicated that anthocyanins are rapidly and thoroughly metabolized in the gut such that very little of the parent compounds are able to be absorbed in their native state [167,168,169]. Even when injected directly into blood, anthocyanins are rapidly distributed to various organs, including the brain, and undergo extensive biotransformation and degradation, with some estimates suggesting that total body clearance of a single anthocyanin dose may occur in as little as an hour [18]. This has led to the hypothesis that anthocyanin metabolites are likely to mediate many of the biological effects observed following anthocyanin ingestion in vivo. Thus, it is of great import to investigate the therapeutic properties of these metabolites in order to better understand their potential role in attenuating neurodegenerative disease.

### 3.1. Metabolism, Absorption and Bioavailability of Anthocyanin Metabolites

Following consumption, anthocyanins are detected systemically in several forms, including sulfated, methylated, glucuronidated, and glycosylated conjugates [170,171]. Additionally, metabolism by gut microflora has been shown to produce considerable amounts of phenolic acid and aldehyde metabolites that share many structural characteristics with their respective parent anthocyanins [167,168,169]. These metabolites are often more stable than parent anthocyanins supporting the idea that they are likely the bioactive components of an anthocyanin-rich diet [10,11]. Moreover, recent studies suggest that chronic supplementation with anthocyanin-rich foods, such as strawberries, results in persistently elevated concentrations of several phenolic acid metabolites in the blood of healthy older adults [13].

**Figure 8:**
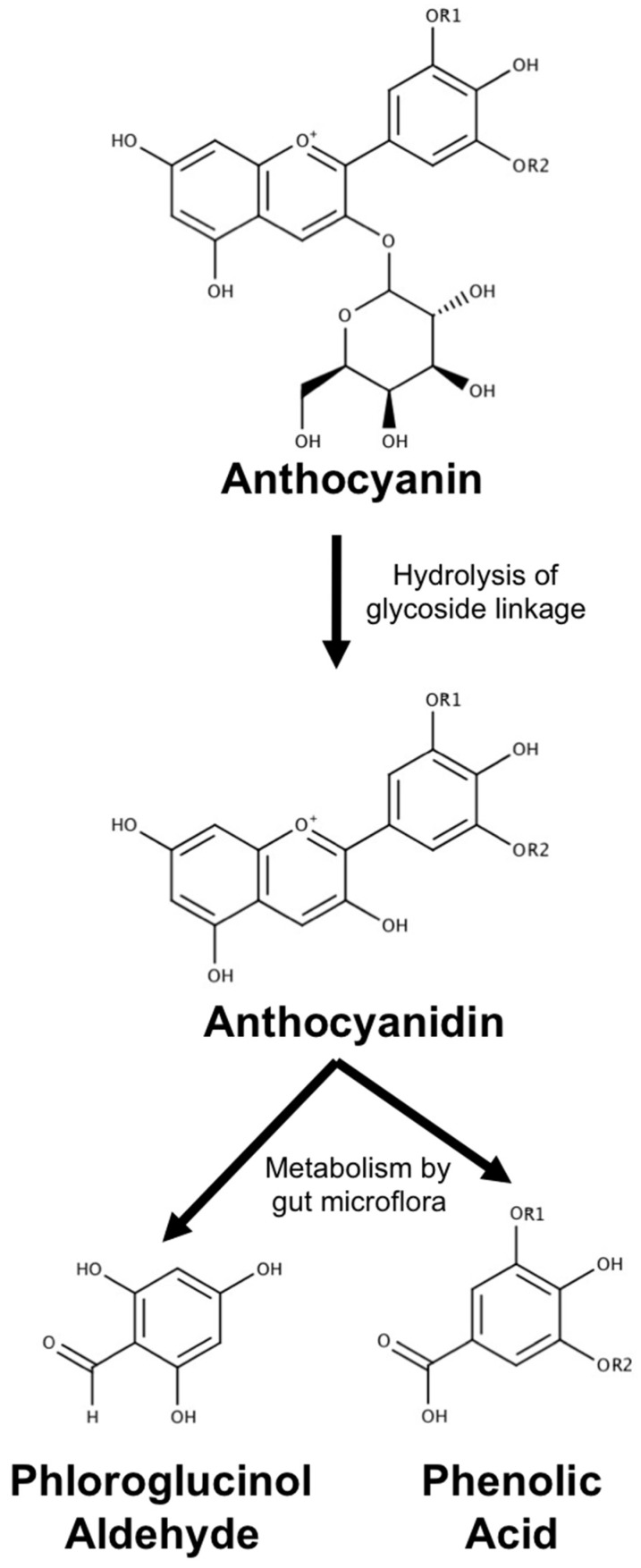
Metabolism of anthocyanins. A generic anthocyanin with a glucoside moiety is pictured. Parent anthocyanin species are first converted to an aglycon (anthocyanidin) form by hydrolysis of glycoside linkages in the small intestine. Upon entry into the large intestine, the anthocyanidin is further metabolized by gut microflora to produce a universal aldehyde metabolite, phloroglucinol aldehyde, and a phenolic acid that retains the structure of the B-ring of the parent anthocyanin.

**Figure 9:**
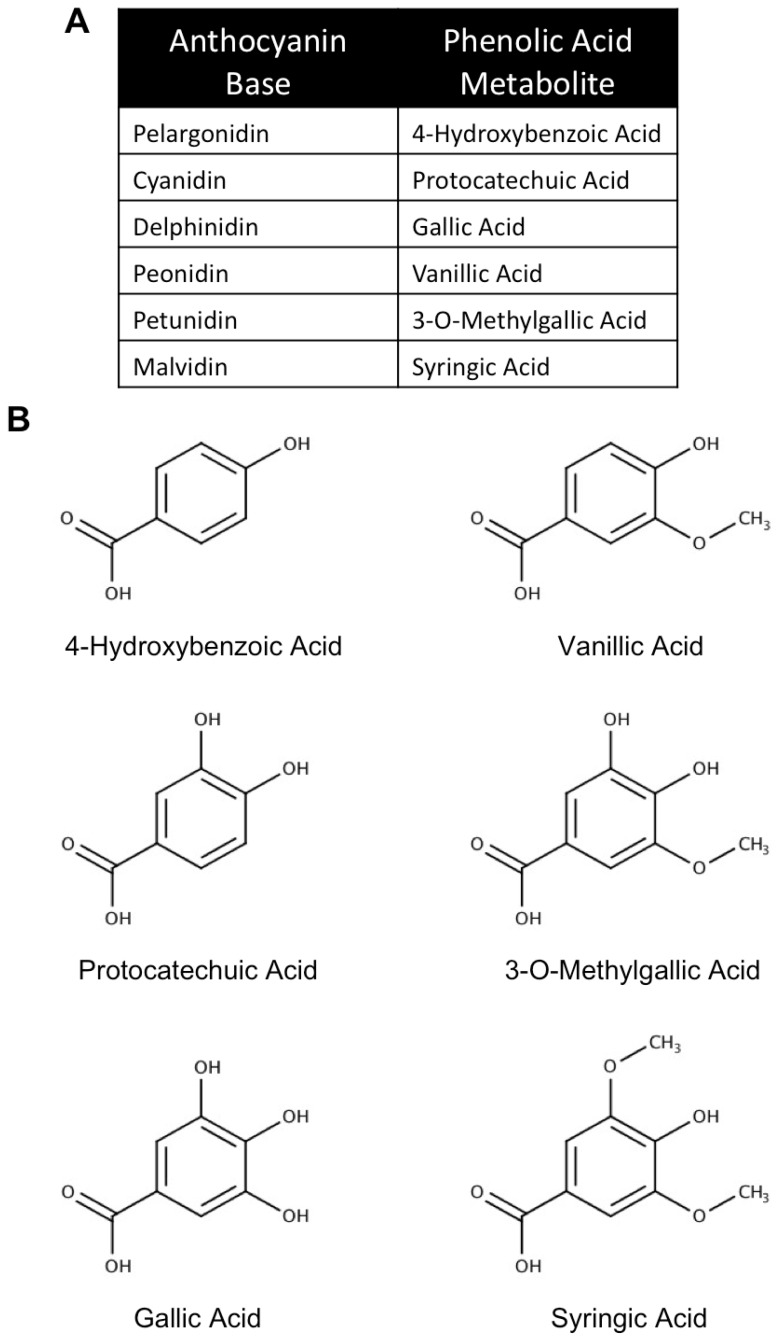
Common phenolic acid metabolites derived from anthocyanins. (**A**) Common anthocyanin bases and their phenolic acid metabolites. (**B**) Molecular structures of six common phenolic acid metabolites derived from anthocyanins.

It is generally accepted that anthocyanins and related polyphenols begin metabolism in the small intestine where glycoside linkages are hydrolyzed to produce the aglycon form [172,173]. Anthocyanins and aglycons that are not absorbed in the small intestine then pass on to the colon, where they are further metabolized by the resident microflora. Metabolism by gut microflora is perhaps the best characterized method by which anthocyanins are degraded and has been described by several studies. In this paradigm, incubation with intestinal microflora has been reported to lead to almost complete degradation of anthocyanins, resulting in the formation of phenolic acids and a universal aldehyde metabolite (Figure 8) [167,168,169]. The phenolic acids derived from this process are the result of hydrolysis of the B-ring and largely retain their structure after separation from the anthocyanin skeleton (Figure 9). Phenolic acid metabolites can be further modified by glucuronidation; however, unaltered phenolic acids have been observed in vivo following anthocyanin ingestion [174]. In particular, the phenolic acid metabolite of cyanidin-based anthocyanins, protocatechuic acid (PCA), has been found in circulation, achieving a concentration eight times higher than that of parent anthocyanins in rat plasma [12]. Interestingly, PCA also appears to remain in tissue longer than its parent anthocyanin [175].

While high circulating concentrations of some metabolites suggest that they may play a significant role in mediating the various health benefits of anthocyanin consumption, it is currently unknown if all anthocyanin metabolites are able to cross the BBB to be absorbed by relevant CNS tissues. However, a growing pool of evidence suggests that phenolic acids are indeed able to gain access to the CNS. One study has reported that PCA can be detected in cortical tissue from rats after oral administration of Danshen extract [176]. Gallic acid has also been detected in brain tissue following oral treatment with grape seed extract, and appears to accumulate in plasma and brain tissue with chronic dosing, strongly suggesting that these compounds are capable of crossing the BBB [177]. Nevertheless, further exploration of this topic is required to assess the therapeutic efficacy of anthocyanin metabolites in vivo.

### 3.2. In Vitro Neuroprotective Effects of Anthocyanin Metabolites

To date, studies assessing the neuroprotective effects of anthocyanin metabolites in vitro, particularly in primary cell cultures, are somewhat limited; however, the few studies that have been conducted show encouraging results for the use of these compounds in mitigating several factors associated with neuronal death in neurodegeneration. The large majority of these studies have examined the beneficial effects of the compounds PCA, gallic acid (GA), and vanillic acid (VA), the phenolic acid metabolites of cyanidin-, delphinidin-, and peonidin-based anthocyanins respectively (Figure 9) [167,168,169]. Limited evidence has also been reported for other metabolites.

Several studies have emerged suggesting that the phenolic acid metabolites of anthocyanins possess pronounced antioxidant capabilities that mediate their neuroprotective effects. For example, treatment of neuronal cell lines with hydrogen peroxide induced a significant degree of cell death, which was entirely attenuated by treatment with PCA [50,178,179,180,181]. This effect was likely mediated in part by the intrinsic free radical scavenging abilities of PCA; however, it was also reported that PCA-treated PC12 cells challenged with hydrogen peroxide possessed higher levels of GSH and catalase activity, suggesting that this compound may also regulate intrinsic antioxidant defenses within the cell [180]. Similar results were observed in PC12 cells and primary cerebellar granule neurons treated with the nitric oxide donor, SNP, with PCA preserving cellular viability to a significant degree [178,179].

GA appears to have similar neuroprotective effects. Indeed, GA treatment significantly attenuated increased calcium levels in addition to mitigating ROS accumulation and preventing the induction of pro-inflammatory enzymes such as COX-2 and p38-MAPK in PC12 cells treated with kainic acid [182]. Similar results were observed in primary cortical neurons challenged with glutamate in which it was shown that GA treatment significantly attenuated cell death by preserving GSH levels and enhancing the activity of SOD and catalase, while decreasing expression of inflammatory cytokines [183].

VA has also been shown to have significant antioxidant capabilities. An extract derived from *Aphanizomenon flos-aquae* cyanobacteria, of which VA is a major component, significantly protected a neuroblastoma cell line from tert-butylhydroperoxide-induced oxidative stress [184]. Moreover VA was shown to significantly reduce oxidative stress induced by hydrogen peroxide and preserve cellular viability in the SH-SY5Y neuronal cell line through elevated expression of several antioxidant enzymes and pro-survival signaling pathways [185]. Similar results were also shown for extracts from white and gold sesame seeds, and pure VA, which were observed to protect SH-SY5Y cells from oxidative damage induced by peroxyl radicals and apoptosis induced by camptothecin [186,187]. Extracts from jucara fruit, which are composed predominately of VA, have also been shown to protect the HT22 hippocampal neuron cell line from toxicity induced by glutamate [188].

4-hydroxybenzoic acid, the metabolite of pelargonidin-based anthocyanins, has also been found to be neuroprotective against hydrogen peroxide-induced oxidative stress and glutamate excitotoxicity, suggesting that this metabolite may also possess significant antioxidant abilities in vitro [178]. One study also reported that syringic acid is protective against hydrogen peroxide toxicity in a retinal ganglion cell line and that these effects were dependent upon the ability of this compound to prevent oxidative stress while activating pro-survival signaling through the PI3K/Akt and Bcl-2 pathways, and decreasing pro-apoptotic signaling [189]. Positive findings were made in primary hippocampal cells treated with syringic acid and exposed to oxygen-glucose deprivation and reperfusion, a cellular model of ischemic injury and stroke that shares many pathological features with neurodegenerative disease [190]. In this work, it was noted that treatment with syringic acid preserved cellular viability, increased levels of antioxidant enzymes, prevented increases in intracellular calcium, and decreased pro-apoptotic signaling through the JNK and p38-MAPK pathways [190].

PCA has also been examined for its ability to attenuate neuronal death induced by treatment with various chemicals associated with models of Parkinson’s disease, such as the complex I inhibitor, MPP^+^. These studies have indicated that PCA effectively protects PC12 cells from MPP^+^-induced toxicity by preventing mitochondrial dysfunction and elevating levels of antioxidant enzymes such as catalase, SOD1, and glutathione peroxidase, in addition to enhancing levels of the pro-survival protein, Bcl-2 [179,191,192]. Similar results were observed when this study was repeated using rotenone, another inhibitor of complex I [193]. The effect of PCA on 6-OHDA toxicity has also been evaluated in PC12 cells where it was found to preserve cellular viability by enhancing the activity and expression of several antioxidant proteins through the induction of Nrf-2, and decreasing the expression of pro-inflammatory markers, such as NF-κB [194]. Interestingly, these effects were enhanced by co-administration of chrysin, another natural flavonoid that is found along with PCA in the fruits of *Alpinia oxyphylla*, and administration of both PCA and chrysin was required to protect dopaminergic neurons in a zebra fish model of Parkinson’s disease [194]. GA and another anthocyanin metabolite, syringic acid, which is derived from malvidin-based anthocyanins, were also evaluated in vitro for their ability to attenuate 6-OHDA toxicity. This study found that both GA and syringic acid were effective neuroprotective agents in this model, though the mechanism of protection was not well defined [195]. Further study on the mechanism underlying the neuroprotective effects of GA against 6-OHDA in SH-SY5Y cells revealed that GA significantly enhanced expression of Bcl-2 and antioxidant enzymes including Nrf-2, while reducing expression of pro-apoptotic proteins Bax and caspase-3, and Keap1, which inhibits Nrf-2 activity [196]. Additionally, the expression of both CREB and BDNF were enhanced, which may suggest a positive effect of GA on learning and memory pathways [196].

The impact of anthocyanin metabolites has also been evaluated for cellular models of neurotoxicity observed in Alzheimer’s disease. PCA has been shown to mitigate the neurotoxic effects of amyloid beta in primary rat cortical neurons by attenuating increases in ROS and intracellular calcium levels, in addition to preventing glutamate release caused by this insult [197]. Evidence has also accumulated suggesting that GA effectively defends neuronal cells from amyloid beta toxicity in a manner very similar to that of PCA, reducing accumulation of intracellular ROS and calcium, and preventing glutamate release [198,199]. Interestingly, regulation of calcium signaling has also been reported in one study utilizing GA to blunt kainate-induced excitotoxicity in PC12 cells where it significantly attenuated increases in intracellular calcium levels [182]. GA has also been demonstrated to increase calbindin expression, a marker of preserved calcium homeostasis, in primary cortical neurons challenged with glutamate [183]. An extract rich in VA has also shown positive effects in the context of amyloid beta toxicity in a neuroblastoma cell line, preventing oxidative stress, and blunting the inflammatory effects of this insult by decreasing NF-κB activation and inflammatory cytokine expression [184]. These observations were confirmed in HT22 cells treated with amyloid beta and pure VA [200]. Collectively, these studies indicate that PCA, GA, and VA may be effective treatments to regulate disruptions in calcium homeostasis observed in neurodegenerative diseases like Alzheimer’s disease.

GA has also been observed to ablate the neurotoxic effects of microglial inflammation induced by amyloid beta in a co-culture system utilizing either primary microglia or BV2 microglia and the neuro2A cell line [201]. Treatment with GA significantly reduced production of pro-inflammatory cytokines, and induced acetylation of NF-κB, thereby decreasing inflammatory gene expression. This translated into preservation of neuronal viability. Likewise, PCA has also been reported to reduce microglial inflammation in BV-2 cells, significantly decreasing the amount of nitric oxide produced by these cells following treatment with LPS [178]. Additionally, PCA treatment also ablated production of pro-inflammatory cytokines and prostaglandin E2 (PGE2), and significantly decreased the expression and activity of several inflammatory mediators including NF-κB, TLR4, and several MAPKs such as ERK, JNK, and p38-MAPK [202]. Taken together, these results suggest that some anthocyanin metabolites may be effective anti-neuroinflammatory agents.

Intriguingly, both GA and VA were shown to have additional neuroprotective and anti-inflammatory abilities beyond simply preserving cellular viability and reducing inflammatory markers. A recent work suggests that both phenolic acids are capable of stimulating neurite outgrowth in primary hippocampal neurons [203]. Moreover, in mixed primary glial and neuronal cultures, treatment with lysolecithin lysophosphatidyl-choline (LPC) caused extensive demyelination of neurons and oligodendrocytes in addition to promoting expression of inflammatory markers, such as NF-κB, COX-2, and GFAP in astrocytes, which was abrogated by treatment with both GA and VA. Indeed, both GA and VA promoted remyelination, significantly reduced deposition of chondroitin sulfate proteoglycans and tenascins by glial cells, which act as a blockade to neuronal regeneration, and restored neuronal function and firing rate [203]. As axonal damage and inability of neurons to transmit important signals is a significant aspect of most neurodegenerative diseases, these findings have important therapeutic implications for GA and VA.

**Figure 10:**
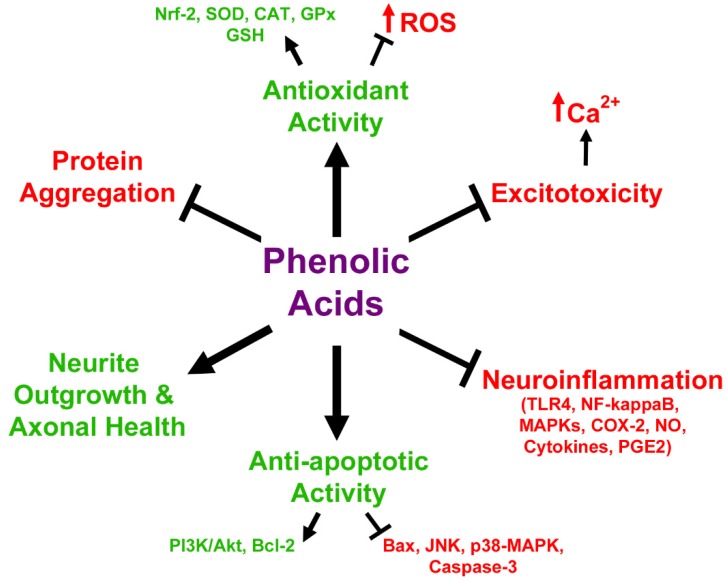
The neuroprotective activities of anthocyanins metabolites. The phenolic acid metabolites display a host of neuroprotective activities. These compounds have been shown to have potent antioxidant abilities through the activation of antioxidant enzymes and direct scavenging of ROS, and are known to prevent excitotoxicity by preserving calcium homeostasis. Additionally, phenolic acids prevent neuroinflammation by reducing the expression of pro-inflammatory pathways in astrocytes and microglia. Many phenolic acids have been shown to directly interfere with aggregation of proteins such as amyloid beta and alpha-synuclein. Moreover, the phenolic acid metabolites of anthocyanins activate pro-survival signaling pathways, while inhibiting expression and activation of pro-apoptotic signaling cascades. Uniquely, these compounds have also been shown to promote neurite outgrowth and axonal health, which may preserve important signaling networks in the brain and spinal cord.

There is also a growing number of reports indicating that anthocyanin metabolites interfere with oligomerization of proteins such as amyloid beta and α-synuclein into toxic fibrils, which are characteristic of Alzheimer’s and Parkinson’s disease pathology, respectively. GA was shown to interfere with amyloid beta fibril formation in primary rat hippocampal cells, which was thought to contribute to its protective effects against amyloid beta toxicity [199]. These results were further corroborated in primary cortical neurons, and further study has revealed that GA may destabilize amyloid beta and prevent its aggregation by interacting directly with the peptide to block sites that allow amyloid beta to form stable oligomeric structures [198]. A similar observation was made for PCA, which both inhibited the oligomerization of amyloid beta, and destabilized pre-formed fibrils, which resulted in enhanced viability in a neuronal cell line [204]. Moreover, a novel composition of palm oil, which is rich in PCA and 4-hydroxybenzoic acid, with lesser amounts of syringic, gallic, and vanillic acids, has been proposed for the treatment of Alzheimer’s disease based on its ability to inhibit amyloid beta fibril formation [205]. Extracts rich in vanillic acid from cyanobacteria and biotransformed black sesame seed pigment have also shown inhibitory activity towards amyloid beta aggregate formation, and were reported to significantly reduce the activity of beta-secretase and prevent the toxicity of such aggregates [184,206]. In a similar fashion, both PCA and GA were found to inhibit the formation of toxic α-synuclein fibrils, destabilize pre-formed fibrils of this protein, and preserve cellular viability in a cell line treated with α-synuclein oligomers and protofibrils [192,204,207]. Though it is not clear how PCA carries out this function, GA was found to inhibit fibril formation by transient interaction with α-synuclein in its extended native structure, which prevented its collapse and subsequent formation of toxic fibrils [207]. Moreover, GA has also been shown to potently inhibit aggregation of proteins induced by perturbations in metal ion levels, which are a hallmark of several neurodegenerative diseases, by acting as a metal chelator to preserve protein homeostasis [208]. While further exploration is needed to determine if these effects can be reproduced with other anthocyanin metabolites, it is a promising finding for their use as therapeutic agents in neurodegeneration for which protein aggregation is a major contributing factor. The neuroprotective activities of the phenolic acid metabolites of anthocyanins are summarized in Figure 10.

### 3.3. Anthocyanin Metabolites as Therapeutic Agents in Neurodegeneration and Aging Models

Studies into the efficacy of anthocyanin metabolites for treating neurodegeneration have gained momentum in the past several years, and many reports have emerged describing the use of these compounds in models of both neurotoxicity and preclinical disease. While the data are sparse and focused almost exclusively around the in vivo neuroprotective effects of PCA, GA, and VA, these initial reports have so far yielded encouraging findings. For example, several studies assessing the effects of phenolic acids against various neurotoxins have been conducted. In a rat model of cadmium-induced heavy metal toxicity, which causes significant disruptions in brain health and cognition, PCA administration was found to significantly elevate expression of antioxidant enzymes, and reduce markers of oxidative stress and lipid peroxidation, as well as decreasing activity of cholinesterases to preserve cholinergic signaling in the brain [209]. Both PCA and GA have also been examined for their ability to mitigate aluminum-induced neurotoxicity in rats, which has been implicated in several neurodegenerative diseases including ALS and Alzheimer’s disease [210,211]. Treatment with PCA and GA relieved deficits in motor and cognitive function respectively in treated animals as well as increasing antioxidant enzymes and decreasing the production of inflammatory cytokines and pro-apoptotic proteins [210,211]. PCA was also reported to inhibit the activity of acetylcholinesterase to a moderate degree as well as that of monoamine oxidases A and B [210]. The ability of GA to ameliorate neurotoxicity induced by quinolinic, an excitotoxic substance that is produced endogenously and that has been implicated in the pathogenesis of neurodegenerative disease, was also recently evaluated [212]. In this model, GA significantly enhanced antioxidant status within the brain and decreased inflammatory and pro-apoptotic markers [212].

The effects of phenolic acids in models of both global and focal ischemia reperfusion have also been the focus of recent study, and provide insight into the neuroprotective and anti-degenerative effects of these compounds. Indeed, PCA was shown to significantly improve cognition in a model of global ischemic injury and was found to enhance neuronal survival in the hippocampus of treated animals [213]. These protective effects were associated with increased levels of GSH, and decreased levels of oxidative damage and astro- and microgliosis [213]. Several reports have shown similarly positive effects on ischemic injury following treatment with VA [214,215,216,217]. These studies indicate that VA treatment also improves cognition and memory in addition to enhancing neuronal viability, expression of antioxidant enzymes, and long-term potentiation [215,216,217]. Moreover, VA treatment has been shown to decrease inflammatory markers such as NF-κB and several pro-inflammatory cytokines within the brain following ischemic injury [216,217]. Notably, both PCA and VA have been shown to preserve the blood–brain barrier, which may contribute to their neuroprotective effects [213,214]. Animals treated with syringic acid displayed similar improvements in markers of injury, showing enhanced neuronal survival and architecture, increased SOD and catalase activity, and decreased expression of caspase-3 and 9 [218]. Collectively, these studies indicate that anthocyanin metabolites possess significant neuroprotective capacities in vivo, which in turn may suggest that they could be effective therapeutic agents for neurodegenerative disorders.

For example, the therapeutic potential of PCA was recently evaluated in the MPTP mouse model of Parkinson’s disease. This study found that treatment with PCA prior to MPTP injection significantly ameliorated the detrimental effects of this insult, preventing loss of tyrosine hydroxylase-positive dopamine neurons in the substantia nigra and preserving levels of dopamine and its metabolites in striatum in comparison to mice receiving MPTP alone [219]. These observations correlated with improved motor function in rotarod testing. Beneficial effects were also observed in this model using an extract from *Sophora tomentosa*, which contains high levels of PCA [220]. In addition to preserving dopaminergic neurons, this extract enhanced antioxidant status in the striatum and significantly reduced the expression of alpha-synuclein and phosphorylated (active) glycogen synthase kinase 3-β (GSK3-β), which can activate pro-apoptotic signaling cascades [220]. Syringic acid was also shown to improve outcomes in the MPTP mouse model by preserving dopaminergic neurons through activation of antioxidant enzymes and reductions in pro-inflammatory markers [221]. Treatment with syringic acid was also protective in this model, and ameliorated reductions in the levels of dopamine and its metabolites as well as that of several enzymes linked to dopamine processing and signaling such as tyrosine hydroxylase, dopamine transporter (DAT), and vesicular monoamine transporter-2 (VMAT-2) [221]. Similar results were reported in a study utilizing GA in the 6-OHDA rat model of Parkinson’s disease. Rats that experienced 6-OHDA lesion displayed dramatic deficits in memory and learning performance measured by passive avoidance testing, which correlated with increased lipid peroxidation and reduced antioxidant defenses in both hippocampus and striatum [222]. These effects were significantly attenuated by GA treatment in a dose-dependent manner. These studies suggest that PCA and GA may be useful for the treatment of Parkinson’s disease and other related disorders.

The therapeutic benefits of PCA, GA and VA treatment were also explored in models of Alzheimer’s disease and aging. In the APP/PS1 mutant mouse model of Alzheimer’s disease, PCA treatment was found to reduce amyloid beta deposition in hippocampal tissue, in addition to reducing expression of APP, from which amyloid beta is generated [223]. PCA also reduced the presence of inflammatory markers in tissue homogenates from hippocampus and cerebral cortex including several inflammatory cytokines such as TNF-α, IL-1β, IL-6, and IL-8, while increasing production of pro-survival signaling factors such as BDNF. Mice that received PCA treatment also displayed marked improvements in learning and memory tasks in comparison to untreated APP/PS1 mice [223]. GA treatment showed similar benefits in this mouse model, producing significant improvements in learning and memory behaviors that were correlated with enhanced long-term potentiation in the hippocampus, and increased dendritic spine density, consistent with improvements in synaptic plasticity [198]. Moreover, GA treatment decreased the size of amyloid plaques within the hippocampus [198]. Several studies in another mouse model of Alzheimer’s disease expressing APPsw demonstrated that extract from date palm fruit, which contains high levels of PCA, improves cognition and decreases levels of amyloid beta protein in the brains of treated mice in addition to reducing levels of oxidative stress and pro-inflammatory cytokines [133,224,225]. Moreover, date palm fruit extract reduced acetylcholinesterase activity, improved the activity of antioxidant enzymes such as SOD and catalase, and preserved GSH levels by enhancing the activity of glutathione peroxidase and glutathione reductase [224]. GA has been shown to have similar effects in mice treated with amyloid beta by intracerebroventricular injection. Amyloid beta injection caused significant impairments in learning and memory tasks in mice, in addition to enhancing pro-inflammatory markers including iNOS and COX-2 expression and production of IL-1β [201]. NF-κB acetylation and nuclear translocation were also examined and found to be significantly enhanced in this model. Pre-treatment with GA, however, potently inhibited these effects, likely through suppression of NF-κB activity [201]. More recently, GA was also shown to improve long-term potentiation and decrease hippocampal amyloid plaque burden rats injected with amyloid beta, which may partially explain its ability to preserve learning and memory functions [226]. VA also attenuates cognitive deficits induced by intracerebroventricular injection of amyloid beta in mice by preserving synapses and decreasing markers of inflammation, oxidative stress, and apoptosis, and reducing amyloid plaque deposition in the hippocampus [200]. Moreover, VA was found to inhibit beta-secretase activity and improved antioxidant status through elevated expression of Nrf-2 [200]. The capacities of GA, and VA have also been examined in a model of streptozotocin-induced dementia. In this context, these phenolic acids were shown to significantly improve antioxidant defenses and decrease markers of oxidative stress in the brain in addition to attenuating the release of pro-inflammatory cytokines [227,228]. VA was also shown to decrease acetylcholinesterase activity in this model, and treatment with this compound was found to improve cognitive behaviors in treated rats [228].

Phenolic acids have additionally been examined for their efficacy in attenuating the effects of age-related decline in the brain. Indeed, in a study utilizing the d-galactose mouse model of aging, PCA administration decreased levels of pro-inflammatory cytokines and reduced COX-2 and NF-κB expression. Moreover, PCA treatment was also reported to reduce oxidative damage in the brains of treated mice [229]. Similar findings were made in the same model when an extract from black mulberry leaves, which is rich in VA, was administered to artificially aged mice [230]. Mice receiving this extract displayed elevated activity of antioxidant enzymes, such as SOD, catalase, and glutathione peroxidase, as well as decreased levels of acetylcholinesterase activity and improved learning and memory [230]. These in vivo results support the assertion that phenolic acid metabolites of anthocyanins may be good candidates for the therapeutic treatment of Alzheimer’s disease and age-related cognitive dysfunction. Nonetheless, further exploration is needed to fully understand the possible therapeutic value of phenolic acid metabolites for various neurodegenerative diseases.

## 4. Future Considerations

While the evidence presented here highlights the great promise of anthocyanins and their metabolites as therapeutic agents for the treatment of neurodegenerative diseases, several considerations must be taken into account to truly understand the safety and efficacy of these compounds for clinical use. Chief among these is the lack of evidence supporting anthocyanins and their phenolic acid metabolites at clinically relevant time points. The vast majority of studies conducted with these compounds have been done in either cell culture or preclinical disease models by pre-treating cells or animals with anthocyanins or their metabolites before administration of a toxic insult or onset of disease symptoms. While these studies are valuable for elucidating the mechanism of action behind the benefits of anthocyanin and phenolic acid administration, they fail to take into account the fact that most cases of neurodegenerative disease are not treated in the clinic until after symptoms have begun to manifest. At this point in the disease course, significant loss of neuronal populations has often already occurred, thus the therapeutic potential of agents with a neuroprotective, rather than a neurorestorative, profile may be limited. As such, further study evaluating the beneficial effects of anthocyanins and their metabolites at or after disease onset is needed in preclinical disease models before further clinical evaluation can take place. Moreover, the use of extracts rich in anthocyanins or their metabolites complicates the interpretation of results as extracts often contain other bioactive compounds that may contribute to improvements in disease state. For this reason, it will be of great interest to expand these positive findings with studies evaluating pure isolates of anthocyanins or their metabolites in preclinical models, and confirm the therapeutic efficacy of these compounds. This may be accomplished with the development of new ways to synthesize pure anthocyanins on a large scale.

Currently, pure anthocyanins, particularly for use in large quantities as in long-term in vivo and human studies, can be cost prohibitive due to the complex and time consuming nature of purification processes. Furthermore, efforts to chemically synthesize anthocyanins have proved challenging, and research to accomplish this feat on an industrial scale is still under development, making anthocyanin extracts from plant material generated in the lab an appealing option for research purposes. While extraction and purification methods for anthocyanins from plants have improved rapidly, these methods still ultimately produce a mixture of anthocyanins rather than pure isolates of single compounds. However, research is now underway to develop novel synthesis methods using metabolically engineered bacteria to generate large quantities of single anthocyanin compounds. Briefly, in this paradigm, recombinant genes encoding enzymes responsible for anthocyanin biosynthesis in plants are introduced into bacteria, and specific chemical precursors to anthocyanins are then added to bacterial cultures to stimulate anthocyanin synthesis, the specific product of which is dependent on the precursor selected. Several studies developing and optimizing this process have been conducted, and strains generating a handful of different anthocyanin species have been described, suggesting that this could be a sustainable and cost effective way to synthesize large quantities of anthocyanins for commercial and research purposes (reviewed by [231]).

Two other considerations include issues of bioavailability and differential activity. As noted before, the bioavailability of anthocyanins is relatively low as these compounds are easily oxidized into less biologically active forms, or rapidly metabolized. For this reason, interest in phenolic acid metabolites as therapeutic agents has grown; however, recent research has also been undertaken to improve both anthocyanin stability and their efficacy in models of neurodegeneration. In particular, encapsulation of anthocyanins in either gold or poly (lactide-*co*-glycolide) (PLGA) nanoparticles conjugated to poly-ethylene glycol (PEG) has shown great potential for improving the therapeutic profile of anthocyanins both in vitro and in Alzheimer’s disease [128,131,135]. When administered to SH-SY5Y cells, PLGA-PEG nanoparticles loaded with anthocyanins were rapidly taken up by cells and shown to enhance the neuroprotective profile of anthocyanins against amyloid beta toxicity above that of anthocyanins alone [128]. Comparable findings were reported for anthocyanins conjugated to PEG-coated gold (PEG-Au) nanoparticles in vitro in BV2 cells [131]. Moreover, PEG-Au nanoparticles loaded with anthocyanins displayed similar activity in vivo in mice that received intracerebroventricular injection of amyloid beta [131,135]. Indeed, the nanoparticles were shown to cross the BBB to deliver anthocyanins to the brain and to improve the neuroprotective, anti-inflammatory, and antioxidant potential of these compounds in comparison to treatment with unconjugated anthocyanins [131,135]. Collectively, these data highlight the importance of anthocyanin stability to their biological activity in the context of neurodegeneration and indicate that methods to preserve anthocyanin structure, such as nanodrug delivery systems, may be a good way to enhance the therapeutic efficacy of these compounds.

It is important to note, however, that not all anthocyanin species possess equal stability and activity. Indeed, anthocyanin structure has been shown to affect their ability to interact with lipid-rich environments and cross in vitro models of the BBB, with more lipophilic anthocyanins preventing lipid peroxidation to a higher degree and crossing the barrier at a higher rate [14,232,233]. Additionally, several reports have also indicated that the neuroprotective activity of both anthocyanins and their metabolites differs based upon their structure [41,51,73,178]. For example, both pelargonidin-3-*O*-glucoside and cyanidin-3-*O*-glucoside are able to protect primary neuronal cells from glutamate toxicity in vitro, but only cyanidin-3-*O*-glucoside is capable of defending these cells from nitric oxide-induced toxicity [41]. Another study noted that the phenolic acid metabolites of these anthocyanins, 4-hydroxybenzoic acid and PCA, also display differential activity based on their structure with both compounds protecting cells from hydrogen peroxide, while only 4-hydroxybenzoic acid protected cells from glutamate excitotoxicity, and only PCA protected neuronal cells from nitric oxide toxicity and reduced LPS-induced inflammation in BV-2 microglial cells [178]. Similarly, delphinidin-based anthocyanins were shown to degrade more rapidly than other anthocyanin species in vitro, which may reduce their efficacy over longer periods, while pelargonidin-based anthocyanins displayed superior stability; however, when pelargonidin-3-*O*-glucoside, cyanidin-3-*O*-glucoside, and malvidin-3-*O*-glucoside were tested for their ability to defend glial cells from hydrogen peroxide-induced damage, only cyanidin-3-*O*-glucoside, and malvidin-3-*O*-glucoside were shown to have protective effects [51]. Similar observations have also been made for anthocyanin-rich extracts, suggesting that overall anthocyanin composition may potently impact the ability of different dietary interventions to protect the brain from different kinds of damage associated with aging and neurodegeneration [41,73]. Thus, while improvements in drug-delivery mechanisms, such as the use of nanoparticles, may circumvent some issues of anthocyanin stability and bioavailability, it may be equally important to consider which individual anthocyanin or phenolic acid metabolites will display the highest level of protective activities upon delivery to the brain when considering the use of these compounds as therapeutic agents. Further, interest in developing flavonoid compounds into MTDLs or other drug types has grown in recent years; thus, these factors should also be taken into account by those considering the use of anthocyanins as potential drug candidates or as scaffolds for future drug discovery efforts. With these factors in mind, effective monitoring of anthocyanin levels and their metabolites will play an important role in confirming efforts to enhance bioavailability and stability. Most human studies to date have used analysis of urine and blood to evaluate the relative amount of anthocyanins and their metabolites remaining in circulation in the body, and those being eliminated. Given a recent report that levels of anthocyanins in blood plasma correlate linearly with levels of anthocyanins in the brain of rats, future studies could use analysis of plasma from blood samples as a way to indirectly monitor anthocyanin levels in the brain over time [18].

Lastly, while both anthocyanins and their metabolites display many pleiotropic effects and target multiple aspects of disease pathology, it is important to consider the use of these compounds in the context of current and new experimental therapies, in which diet or direct supplementation with these compounds could play a complimentary role in the treatment of neurodegenerative disease. Neurodegenerative diseases are complex, and to date, no single therapy has proven effective for treating disease pathology and symptoms, and no treatments currently exist that are capable of halting or reversing disease progression. Thus, the use of multiple complimentary agents and lifestyle interventions becomes an appealing option for treating neurodegenerative diseases. For example, recent evidence has emerged suggesting that many anthocyanins, their phenolic acid metabolites, and extracts rich in these compounds display significant inhibitory activity against enzymes involved in the regulation of synaptic signaling by several neurotransmitters. These include cholinesterases, which regulate acetylcholine levels, and monoamine oxidases, which modulate signaling by monoamines such as dopamine and serotonin. Inhibitors of cholinesterases and monoamine oxidases have been shown to be therapeutic for Alzheimer’s disease and Parkinson’s disease respectively, and these drugs are used regularly in the clinic to treat symptoms of disease (Reviewed by [234,235]). Anthocyanins and anthocyanin-rich extracts have been shown to inhibit acetylcholinesterase in cell culture, as well as in vivo in rodents under both healthy and pathological conditions [62,138,139,148,236,237,238,239]. These results have been reflected in similar studies with both pure phenolic acids and extracts enriched in phenolic acids, particularly those containing VA, PCA, and syringic acid [209,228,240,241,242]. PCA in particular has been shown to reduce activity of both acetylcholinesterase and butyrylcholinesterase in vivo under neurotoxic conditions induced by cadmium exposure [209]. Similar results have been observed for the activity of anthocyanins on monoamine oxidase inhibition as it has been observed that both pure anthocyanins and their aglycons inhibit both monoamine oxidases A and B in vitro, while anthocyanin-rich extracts have been shown to inhibit monoamine oxidase B in rodent models of artificial aging, and in healthy adults [147,243,244]. PCA has also been shown to inhibit both monoamine oxidases in vitro and in vivo, though it has a greater selectivity for monoamine oxidase B [210,245]. Collectively, these data suggest that although these compounds do not inhibit acetylcholinesterase or monoamine oxidases to the same extent as current therapies available on the market, anthocyanins or their metabolites may have a safer profile as therapeutic agents, and thus could serve a complimentary purpose in enhancing current treatments for Alzheimer’s and Parkinson’s disease either through dietary intervention or as supplements [239,243,245].

Other studies, though limited in number, have also indicated that anthocyanins or their metabolites may display synergistic or additive effects when paired with other forms of treatment to relieve inflammation and protect neurons in the CNS. For instance, pelargonidin-3-*O*-glucoside was shown to have an additive effect with isoflurane in vitro to reduce LPS-induced inflammation in a microglial cell line [83]. In vivo, PCA also showed synergistic effects on cellular survival and health with another phytochemical, chrysin, in vitro and in a zebrafish model of Parkinson’s disease in which both compounds were necessary to preserve dopaminergic neurons within the brain [194]. Lastly, and perhaps most intriguingly, PCA has also been shown to have protective effects on neural progenitor cells, and could play a complimentary role in stem cell therapy for various neurodegenerative diseases. Although widely considered to be one of the most promising ways to cure several neurodegenerative diseases, stem cell therapy is still highly experimental, and even though promising results have been observed in preclinical models and some clinical trials (reviewed by [246]), many concerns related to stem cell survival, proliferation, differentiation, and resistance to a diseased environment have arisen. Recently, however, it was shown that PCA is able to enhance differentiation of rat neural progenitors into neurons in vitro in addition to decreasing cell death in differentiating cell populations of both neurons and glia [247,248]. While PCA modestly decreased the number of cells that differentiated into astrocytes in this paradigm, these studies also reported increases in antioxidant enzymes and decreased levels of reactive oxygen species in all differentiated cell types in the presence of PCA [248]. This suggests that the overall health of the cells derived from this process may be superior to that of cells derived under standard differentiating conditions, which could prevent their death or transition to an inflammatory state, or improve their capacity to supply trophic support when introduced into a diseased environment such as the brain or spinal cord of a patient with neurodegenerative disease. In relation to these studies, a diet enriched in blueberry, a good source of anthocyanins, was shown to improve survival of transplanted embryonic dopamine neurons in the brains of treated rats that had undergone unilateral dopamine depletion, resulting in improved motor behavior when compared to implanted rats on a standard diet [249]. This suggests that not only treatment of transplanted cells, but also treatment of the host with anthocyanins or their metabolites may be a viable and simple way to improve the efficacy of stem cell and cell replacement therapies. While a significant amount of research still remains to be done to explore this possibility for safety and efficacy, when considered altogether, these results show precedent for anthocyanins and their phenolic acid metabolites to be used in conjunction with other therapeutic strategies to treat some of the pathological aspects associated with neurodegenerative disease.

## 5. Conclusions

In sum, anthocyanins and their metabolites represent a unique subset of dietary compounds with a wide range of biological activities. Indeed, the ability of these compounds to modulate multiple aspects of disease pathology including antioxidant pathways, calcium homeostasis, inflammation, protein homeostasis, and the balance of pro-survival and pro-apoptotic signaling makes them uniquely suited for the treatment of neurodegenerative diseases. These qualities, combined with their relative safety and low toxicity as a prolific part of most diets, make them appealing targets for further pre-clinical and clinical development. However, future studies should be sure to consider the limitations of individual compounds and consider the role of anthocyanins or their metabolites in the context of existing therapeutic modalities.

## Figures and Tables

**Table 1:** Protein aggregation in selected neurodegenerative diseases.

Neurodegenerative Disease	Major Constituents of Protein Aggregates	Reference
Alzheimer’s Disease	Amyloid Beta Peptide (from APP)	Glenner and Wong, [93]
		Masters et al. [94]
		Selkoe et al. [95]
	Hyperphosphorylated Tau	Bancher et al. [96]
Parkinson’s Disease	α-synuclein	Baba et al. [97]
ALS	C9orf72	Mori et al. [98]
	FUS	Vance et al. [99]
		Ling et al. [100]
	SOD1	Bruijn et al. [101]
		Bosco et al. [102]
	TDP-43	Johnson et al. [103]
		Mackenzie et al. [104]
